# Supplementary material for: Spatially resolved dendritic integration: towards a functional classification of neurons
Source: PeerJ. 2020 Nov 24;8:e10250. doi: 10.7717/peerj.10250 (PMC7694565; doi:10.7717/peerj.10250)

Figure S1. Firing rate heat maps of all neurons varied over P. The activation rate is held constant, h = 0.1 Hz, and the firing rates were averaged over five trials. The soma is marked by the arrow (left panel). Note the different color bars.

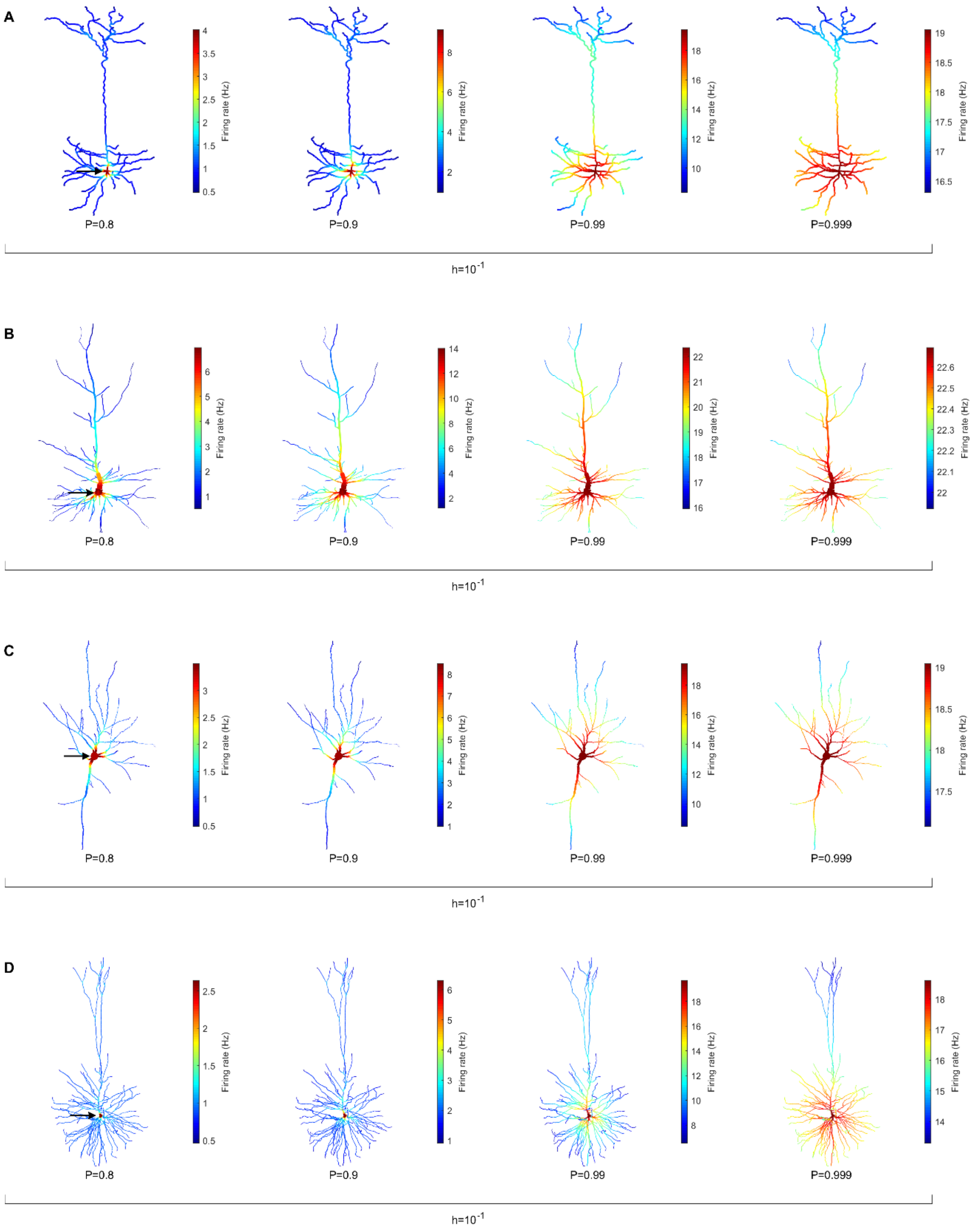

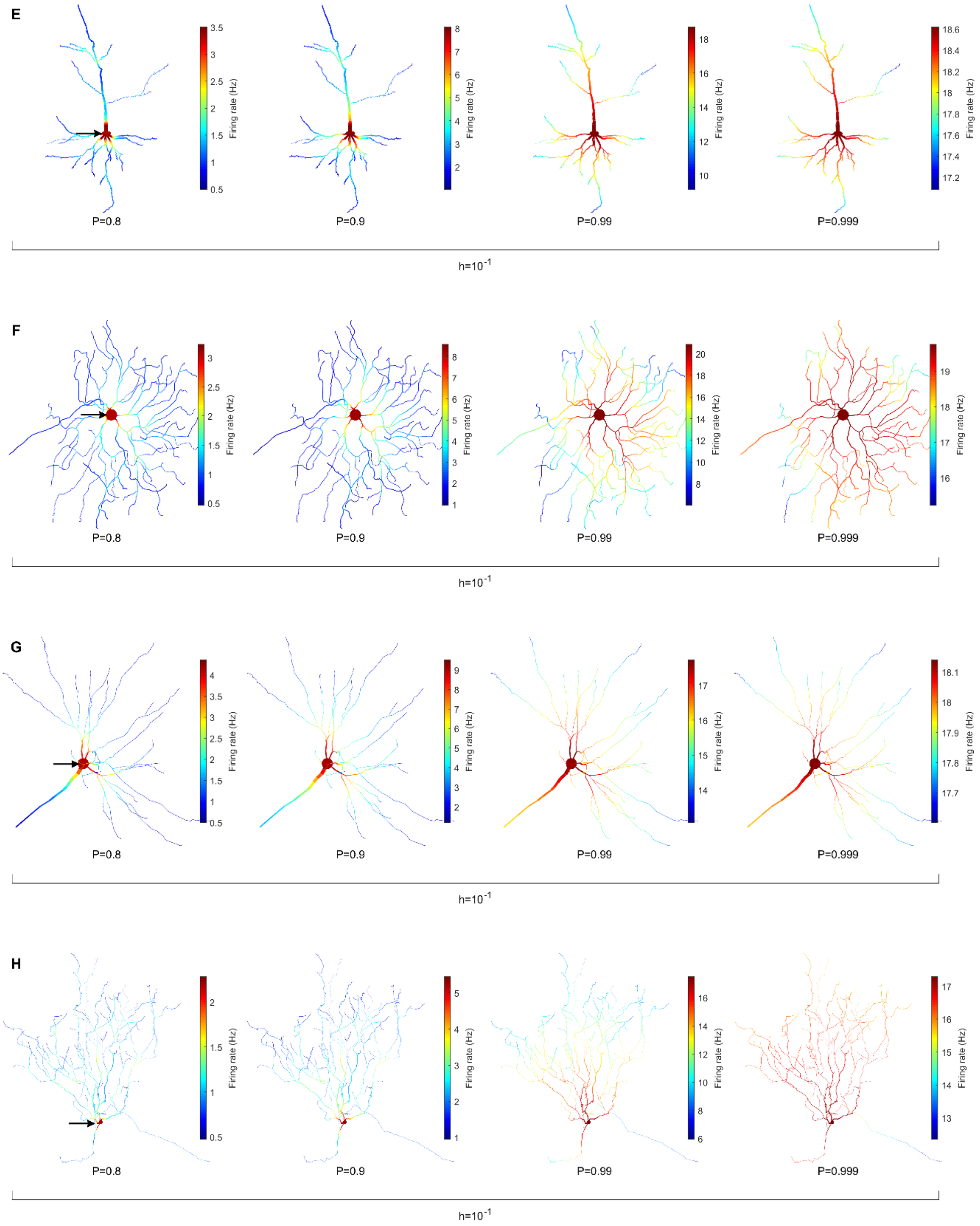
E
P=0.8
P=0.9
P=0.99
P=0.999
h=10^-1
Firing rate (Hz)
F
P=0.8
P=0.9
P=0.99
P=0.999
h=10^-1
Firing rate (Hz)
G
P=0.8
P=0.9
P=0.99
P=0.999
h=10^-1
Firing rate (Hz)
H
P=0.8
P=0.9
P=0.99
P=0.999
h=10^-1
Firing rate (Hz)

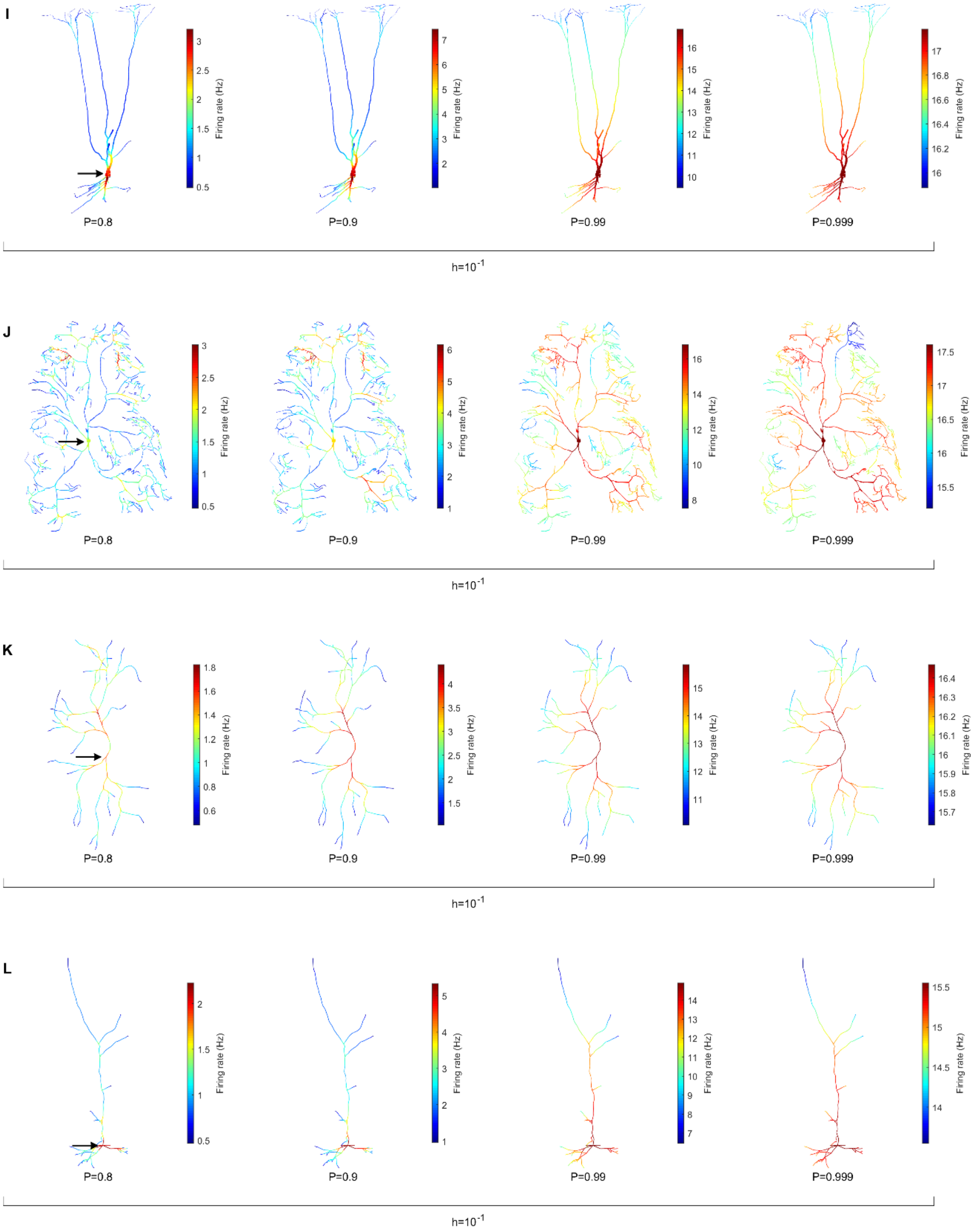
I
Firing rate (Hz)
P=0.8
P=0.9
P=0.99
P=0.999
h=10^-1
J
Firing rate (Hz)
P=0.8
P=0.9
P=0.99
P=0.999
h=10^-1
K
Firing rate (Hz)
P=0.8
P=0.9
P=0.99
P=0.999
h=10^-1
L
Firing rate (Hz)
P=0.8
P=0.9
P=0.99
P=0.999
h=10^-1

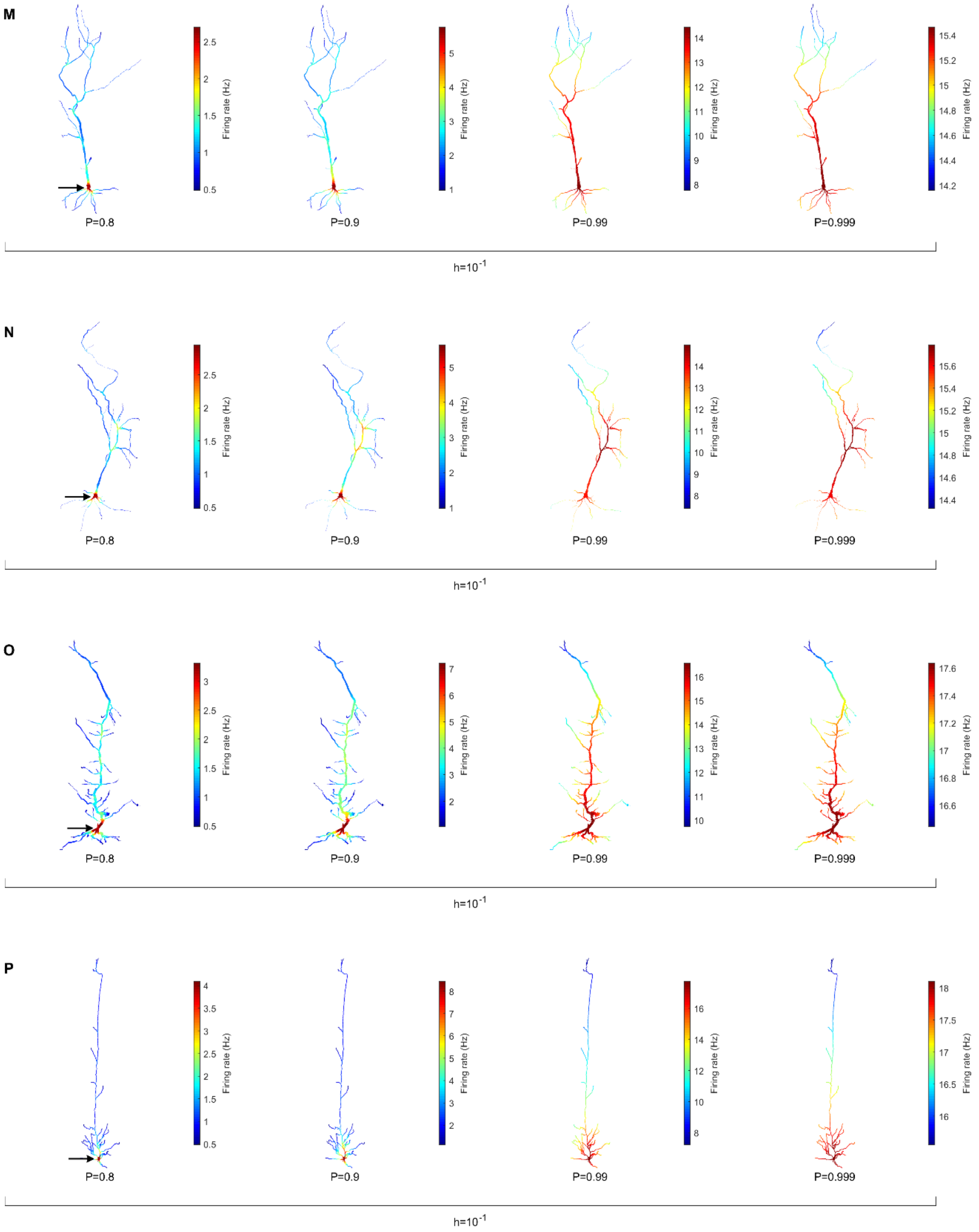
M
N
O
P
P=0.8
P=0.9
P=0.99
P=0.999
h=10^-1
Firing rate (Hz)
0.5 1 1.5 2 2.5
1 2 3 4 5
8 9 10 11 12 13 14
14.2 14.4 14.6 14.8 15 15.2 15.4
0.5 1 1.5 2 2.5
1 2 3 4 5
8 9 10 11 12 13 14
14.4 14.6 14.8 15 15.2 15.4 15.6
0.5 1 1.5 2 2.5 3
2 3 4 5 6 7
10 11 12 13 14 15 16
16.6 16.8 17 17.2 17.4 17.6
0.5 1 1.5 2 2.5 3 3.5 4
2 3 4 5 6 7 8
8 10 12 14 16
16 16.5 17 17.5 18

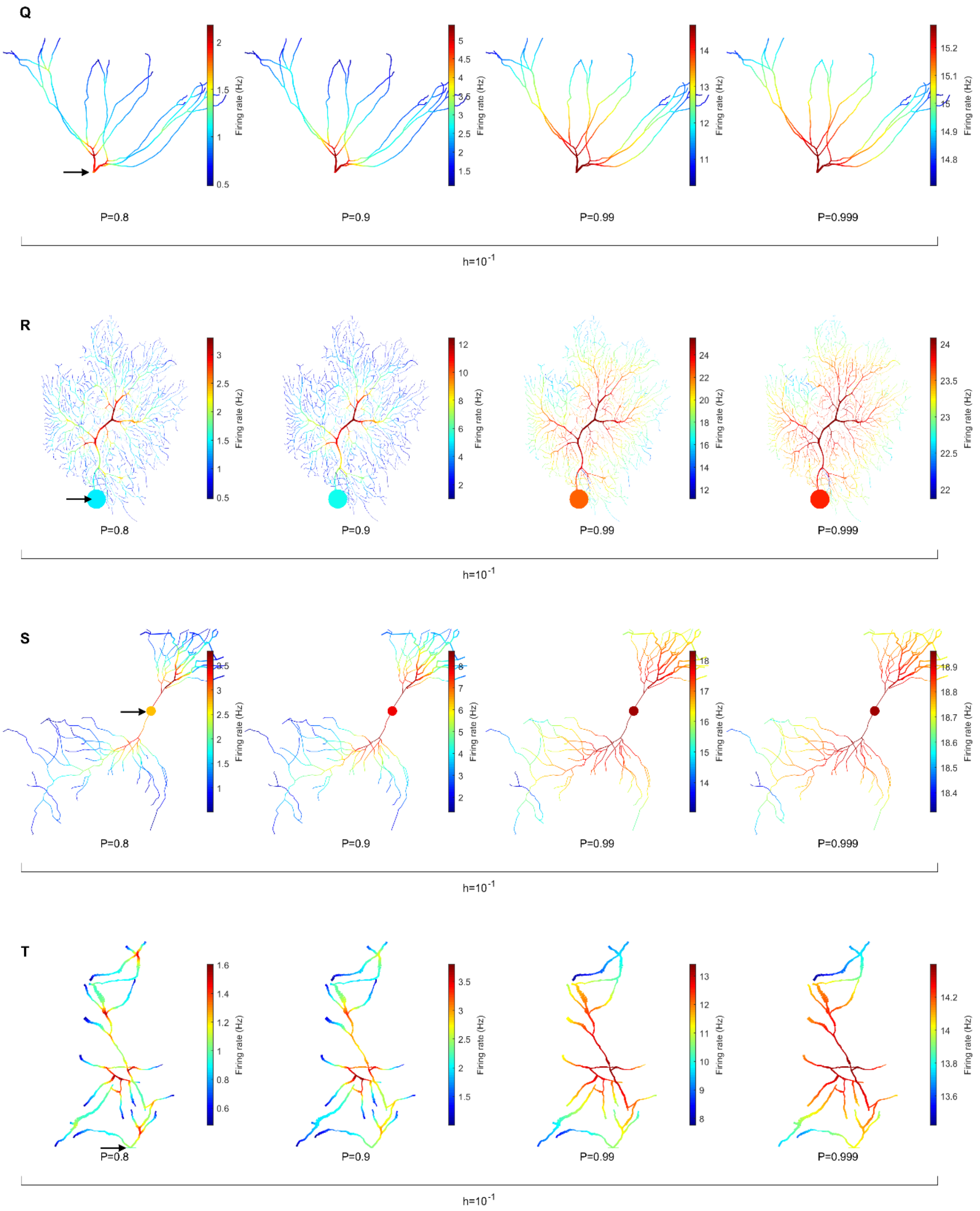
Q
R
S
T
Firing rate (Hz)
P=0.8
P=0.9
P=0.99
P=0.999
h=10^-1

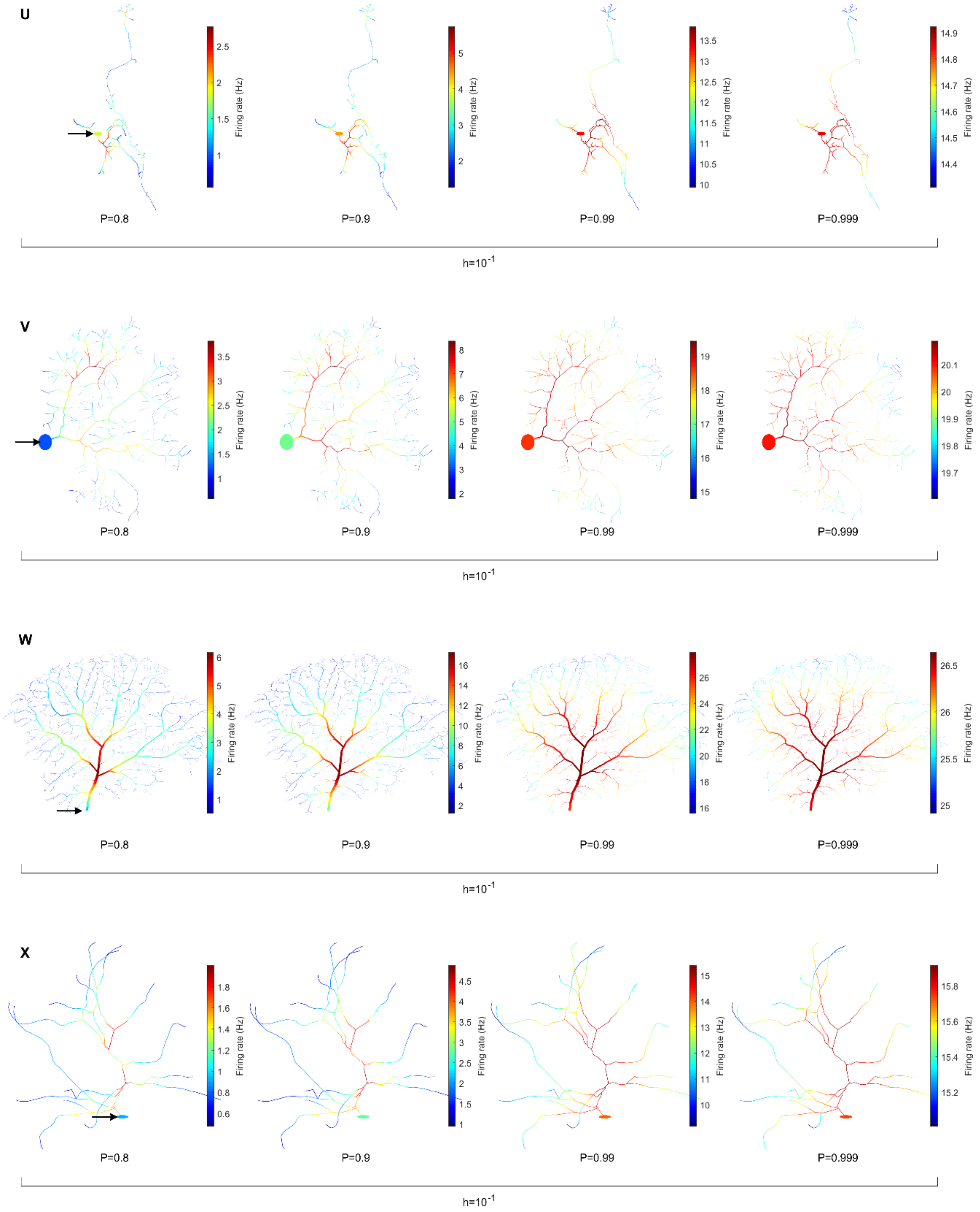
U
2.5
2
1.5
1
Firing rate (Hz)
5
4
3
2
Firing rate (Hz)
13.5
13
12.5
12
11.5
11
10.5
10
Firing rate (Hz)
14.9
14.8
14.7
14.6
14.5
14.4
Firing rate (Hz)
P=0.8
P=0.9
P=0.99
P=0.999
h=10^-1
V
3.5
3
2.5
2
1.5
1
Firing rate (Hz)
8
7
6
5
4
3
2
Firing rate (Hz)
19
18
17
16
15
Firing rate (Hz)
20.1
20
19.9
19.8
19.7
Firing rate (Hz)
P=0.8
P=0.9
P=0.99
P=0.999
h=10^-1
W
6
5
4
3
2
1
Firing rate (Hz)
16
14
12
10
8
6
4
2
Firing rate (Hz)
26
24
22
20
18
16
Firing rate (Hz)
26.5
26
25.5
25
Firing rate (Hz)
P=0.8
P=0.9
P=0.99
P=0.999
h=10^-1
X
1.8
1.6
1.4
1.2
1
0.8
0.6
Firing rate (Hz)
4.5
4
3.5
3
2.5
2
1.5
1
Firing rate (Hz)
15
14
13
12
11
10
Firing rate (Hz)
15.8
15.6
15.4
15.2
Firing rate (Hz)
P=0.8
P=0.9
P=0.99
P=0.999
h=10^-1

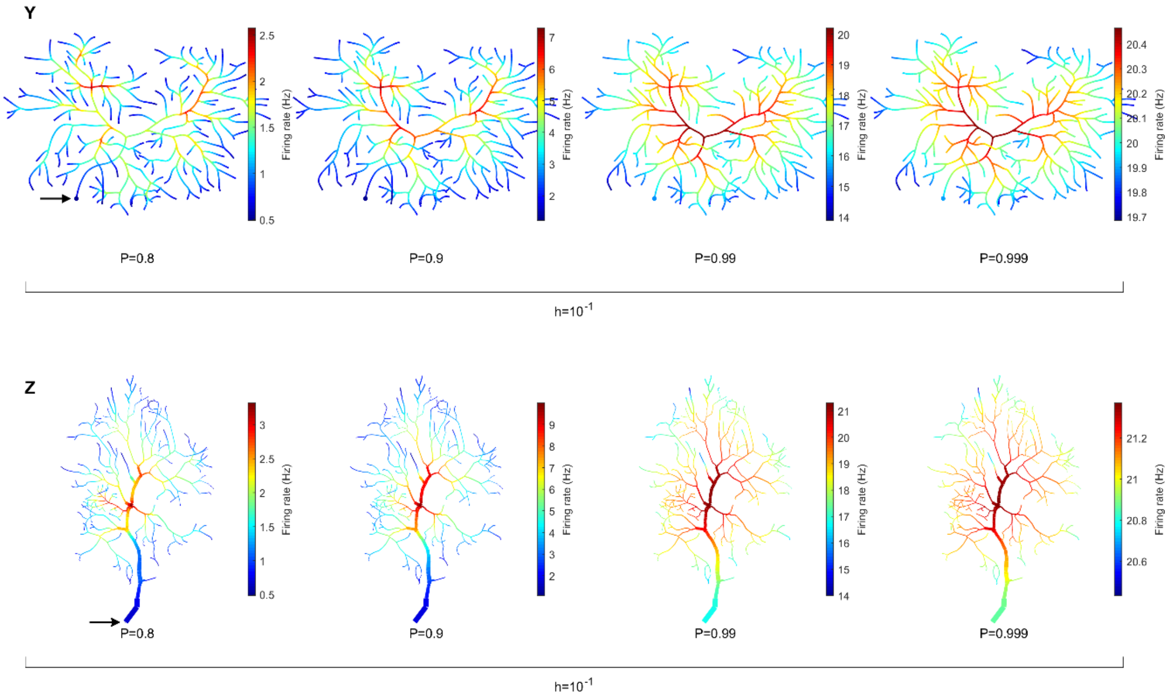

Figure S2. Firing rate heat maps of all neurons varied over h. The transmission probability is held constant, *P* = 0.9, and the firing rates were averaged over five trials. The soma is marked by the arrow. Note the different color bars.

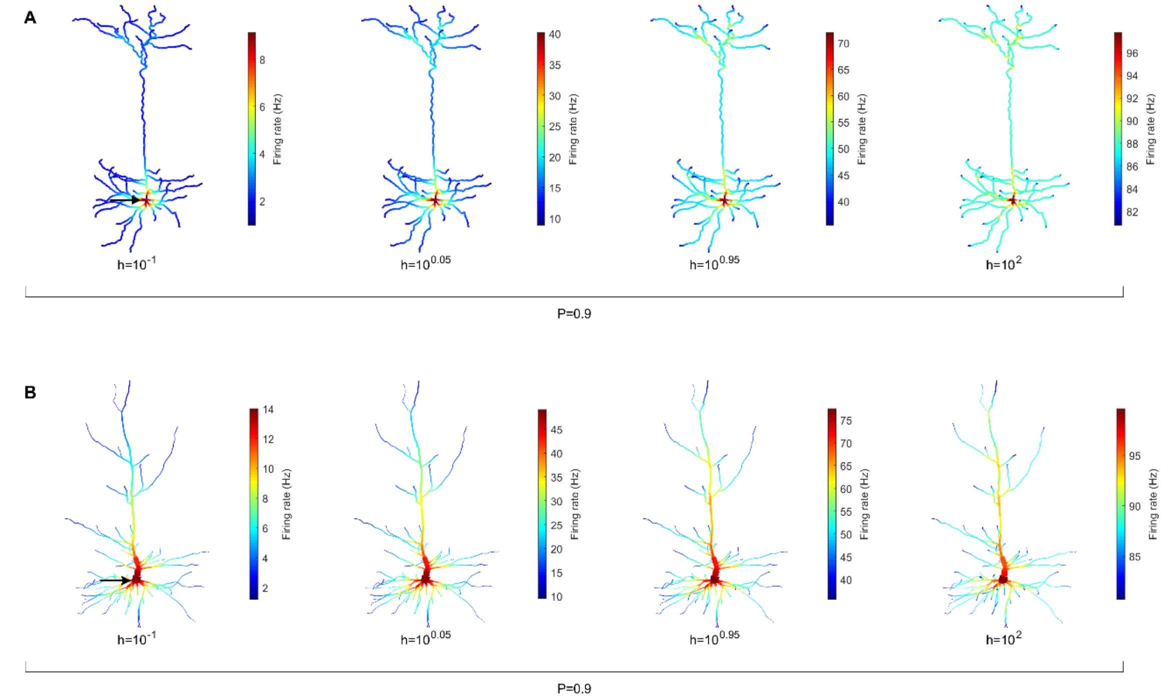

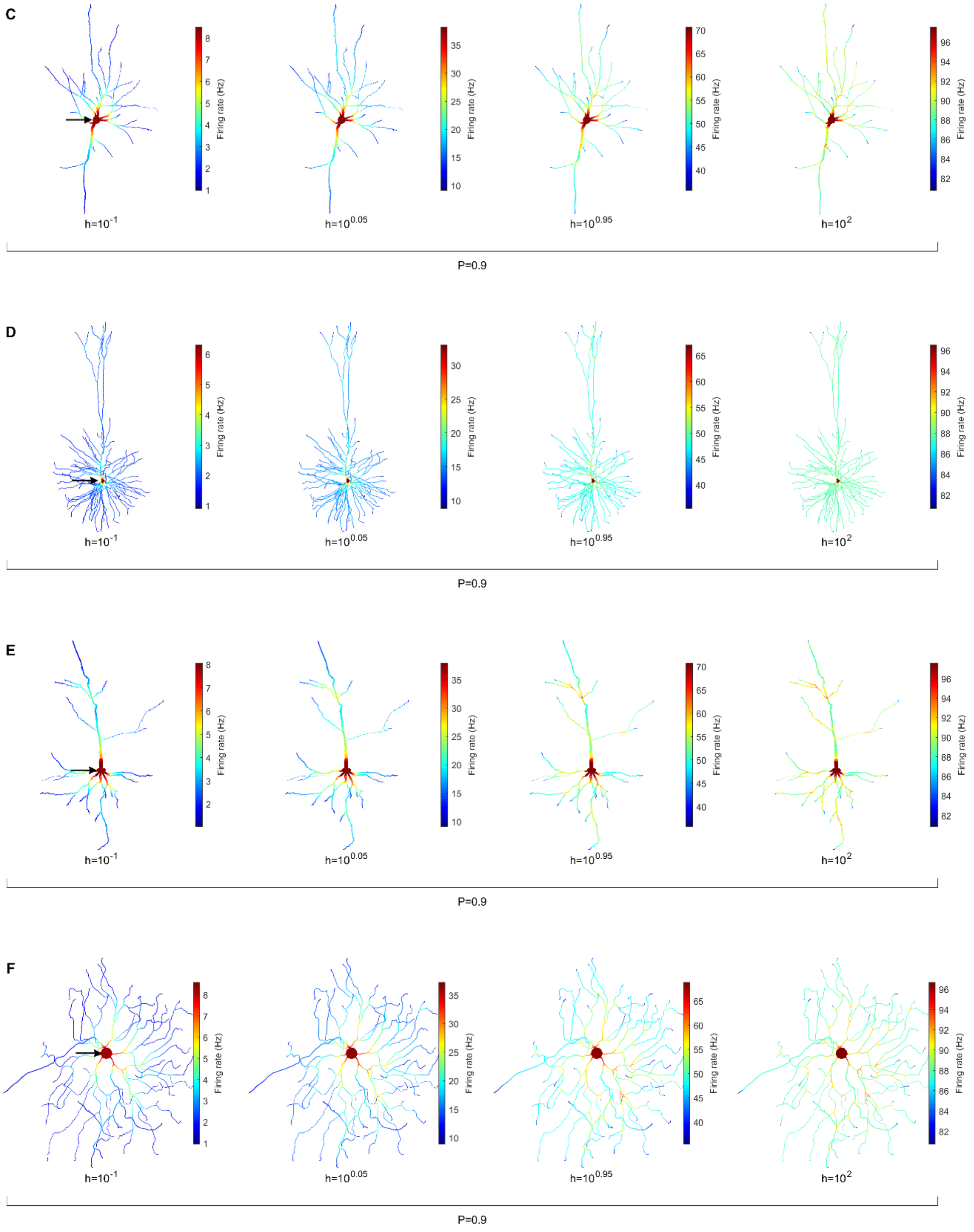

C
Firing rate (Hz)
h=10^{-1}
h=10$^{0.05}$
h=10$^{0.95}$
h=10^{2}
P=0.9
D
Firing rate (Hz)
h=10^{-1}
h=10$^{0.05}$
h=10$^{0.95}$
h=10^{2}
P=0.9
E
Firing rate (Hz)
h=10^{-1}
h=10$^{0.05}$
h=10$^{0.95}$
h=10^{2}
P=0.9
F
Firing rate (Hz)
h=10^{-1}
h=10$^{0.05}$
h=10$^{0.95}$
h=10^{2}
P=0.9

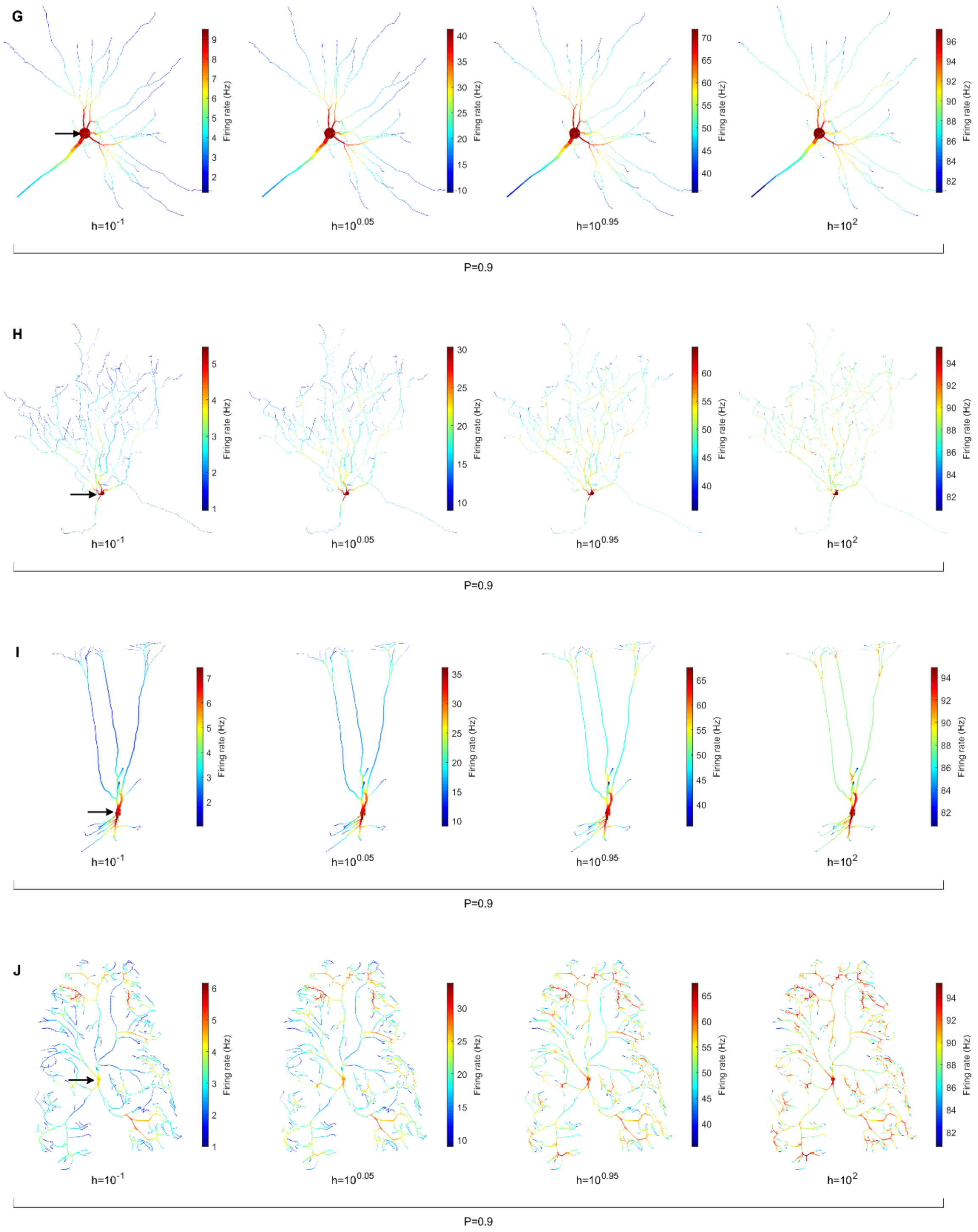

G
Firing rate (Hz)
9
8
7
6
5
4
3
2
40
35
30
25
20
15
10
70
65
60
55
50
45
40
96
94
92
90
88
86
84
82
h=10-1
h=100.05
h=100.95
h=102
P=0.9
H
Firing rate (Hz)
5
4
3
2
1
30
25
20
15
10
60
55
50
45
40
94
92
90
88
86
84
82
h=10-1
h=100.05
h=100.95
h=102
P=0.9
I
Firing rate (Hz)
7
6
5
4
3
2
35
30
25
20
15
10
65
60
55
50
45
40
94
92
90
88
86
84
82
h=10-1
h=100.05
h=100.95
h=102
P=0.9
J
Firing rate (Hz)
6
5
4
3
2
1
30
25
20
15
10
65
60
55
50
45
40
94
92
90
88
86
84
82
h=10-1
h=100.05
h=100.95
h=102
P=0.9

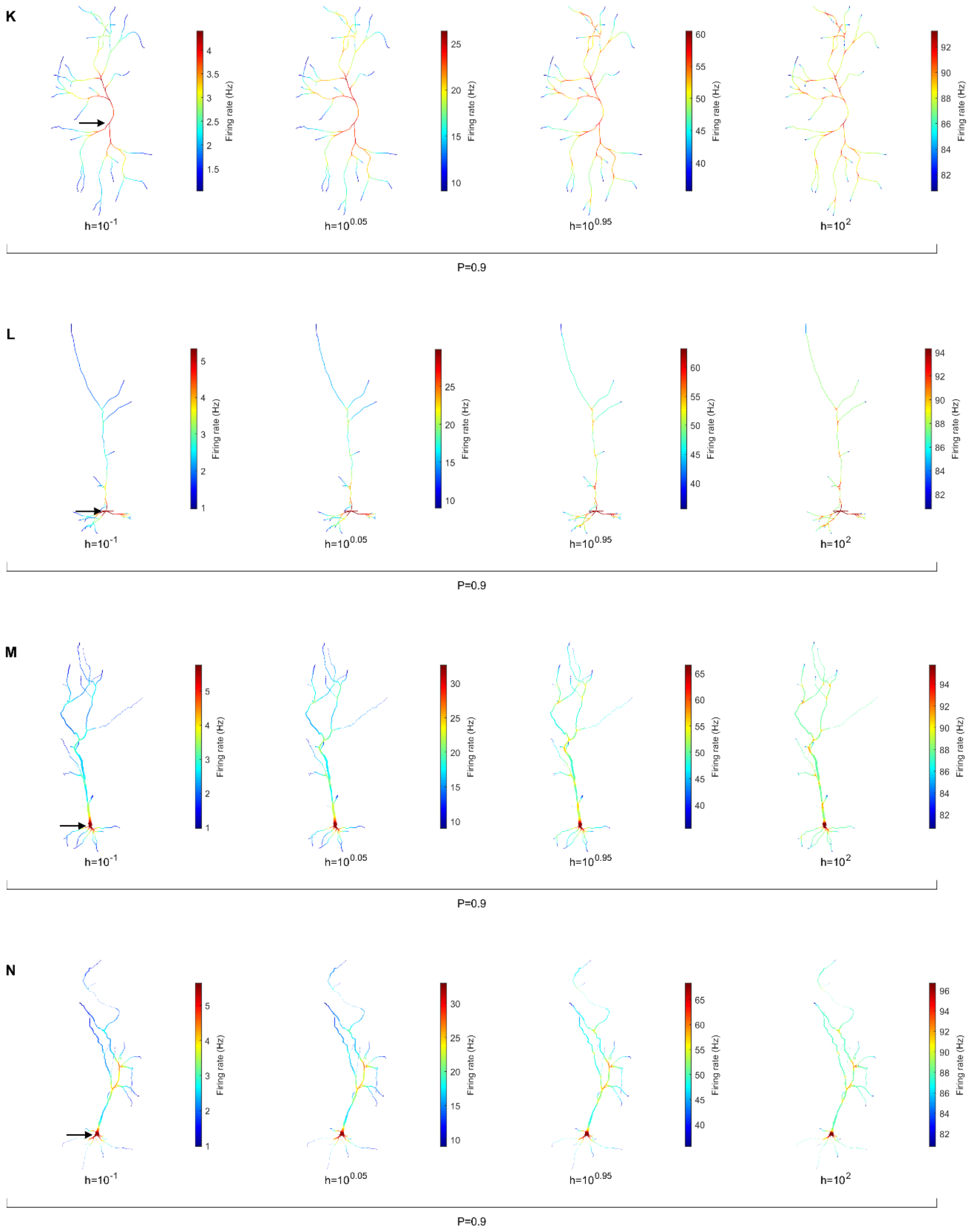
K
L
M
N
Firing rate (Hz)
h=10^-1
h=10^0.05
h=10^0.95
h=10^2
P=0.9

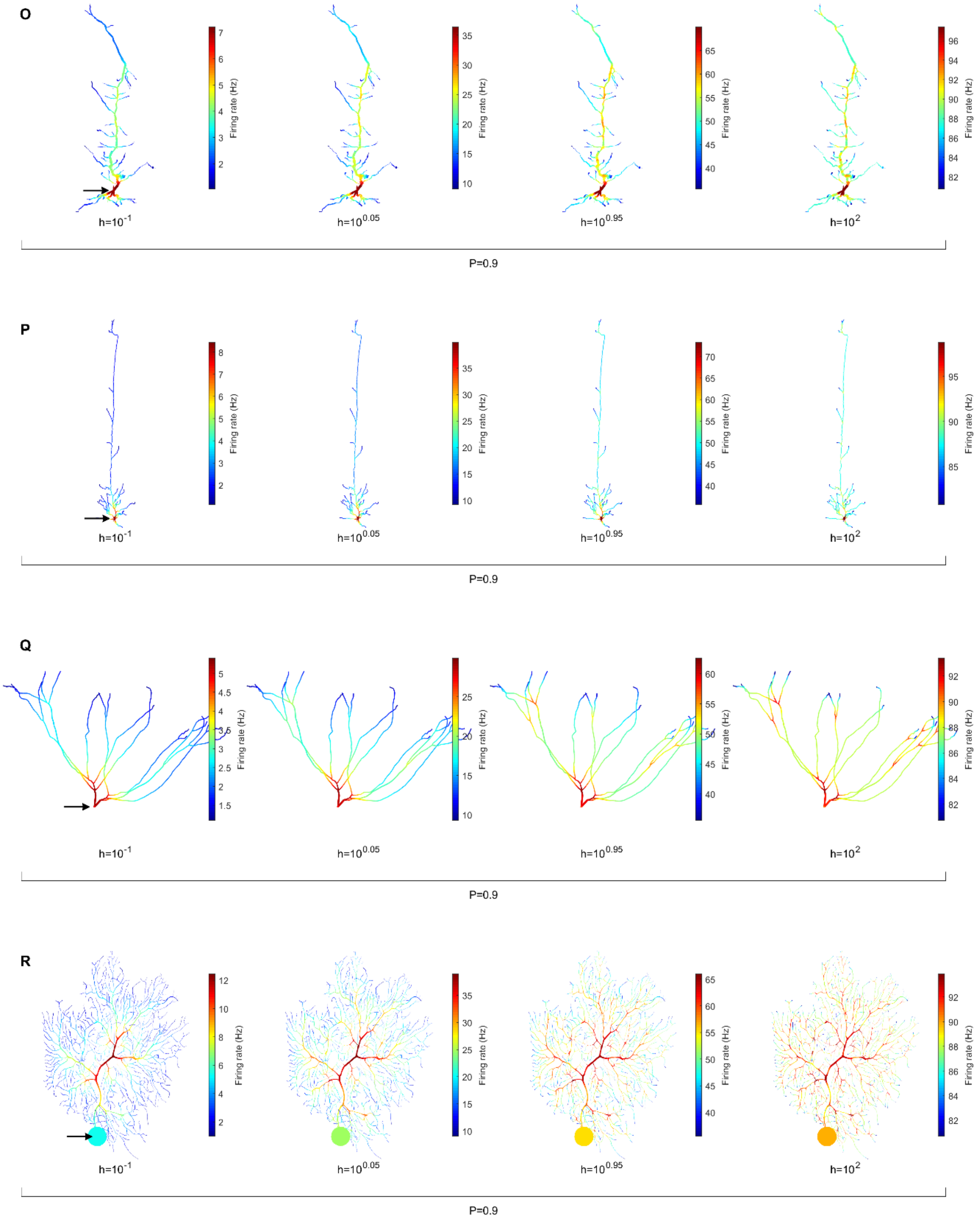
O
h=10-1
h=100.05
h=100.95
h=102
Firing rate (Hz)
P=0.9
P
h=10-1
h=100.05
h=100.95
h=102
Firing rate (Hz)
P=0.9
Q
h=10-1
h=100.05
h=100.95
h=102
Firing rate (Hz)
P=0.9
R
h=10-1
h=100.05
h=100.95
h=102
Firing rate (Hz)
P=0.9

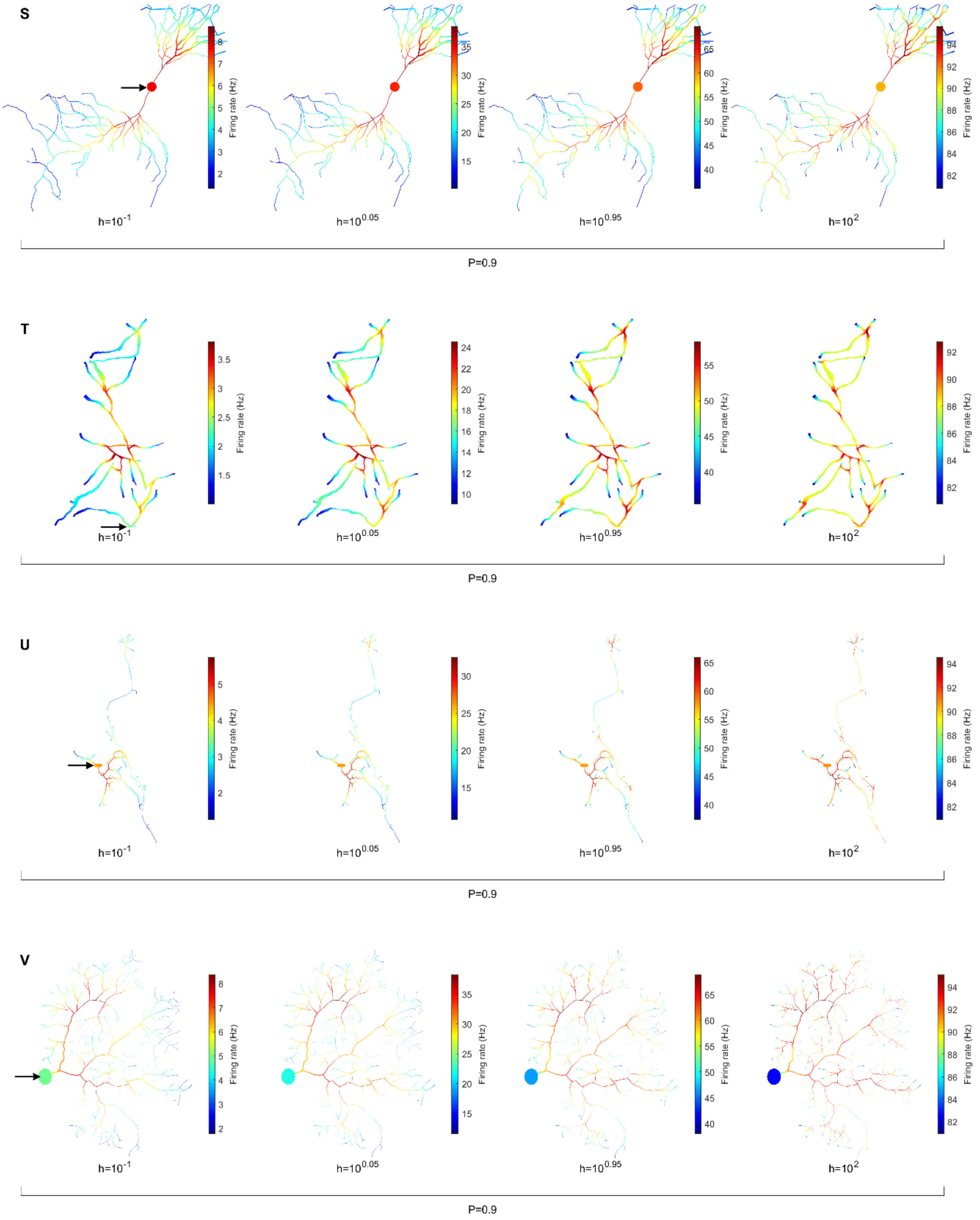

S
Firing rate (Hz)
h=10^{-1}
h=$10^{0.05}$
h=$10^{0.95}$
h=10^{2}
P=0.9
T
Firing rate (Hz)
h=10^{-1}
h=$10^{0.05}$
h=$10^{0.95}$
h=10^{2}
P=0.9
U
Firing rate (Hz)
h=10^{-1}
h=$10^{0.05}$
h=$10^{0.95}$
h=10^{2}
P=0.9
V
Firing rate (Hz)
h=10^{-1}
h=$10^{0.05}$
h=$10^{0.95}$
h=10^{2}
P=0.9

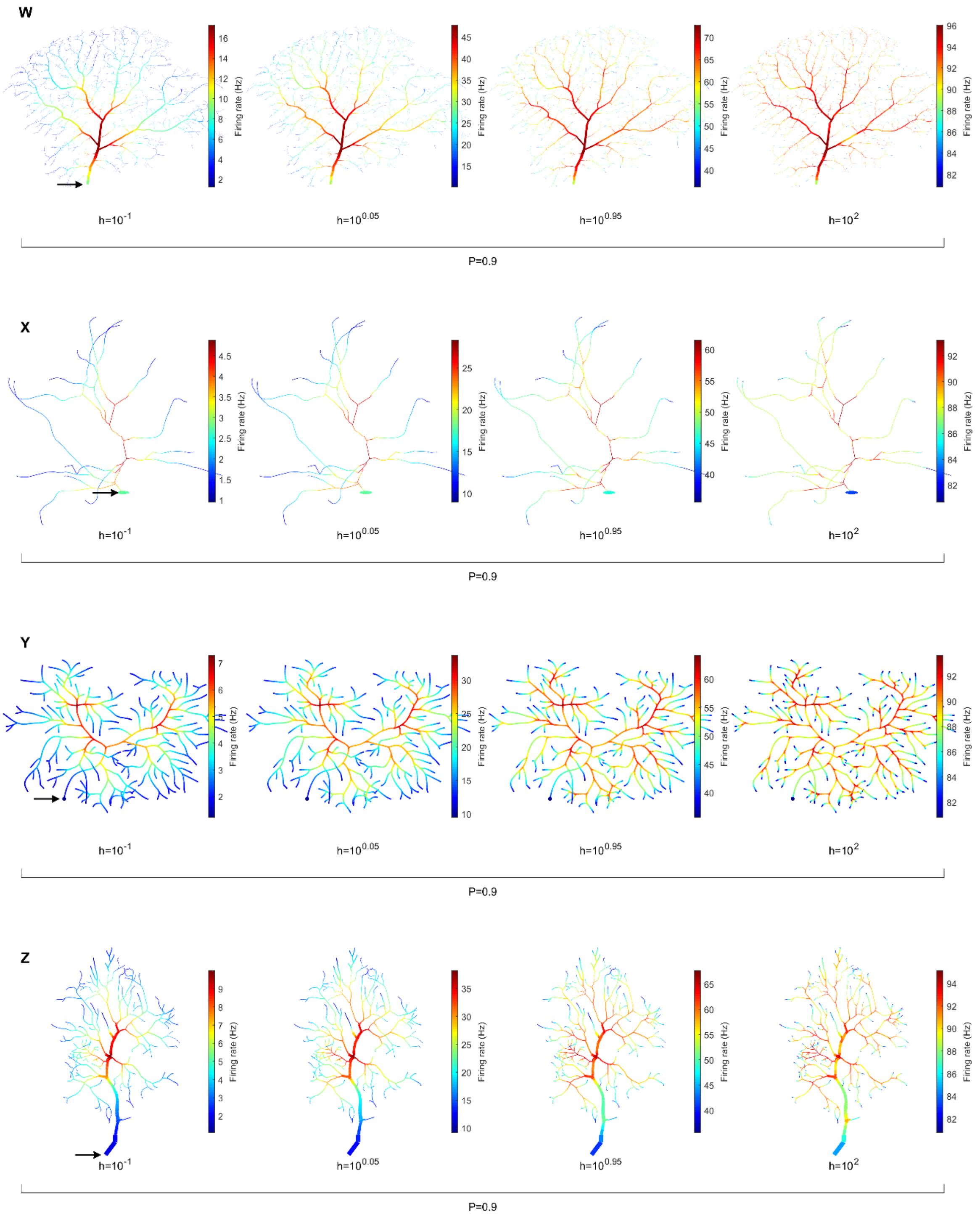
W
h=10^-1
h=10^0.05
h=10^0.95
h=10^2
P=0.9
X
h=10^-1
h=10^0.05
h=10^0.95
h=10^2
P=0.9
Y
h=10^-1
h=10^0.05
h=10^0.95
h=10^2
P=0.9
Z
h=10^-1
h=10^0.05
h=10^0.95
h=10^2
P=0.9
Firing rate (Hz)

Figure S3. Dynamic range heat maps of all neurons varied over P. Values were averaged over five trials. The soma is marked by the arrow. Note the different color bars.

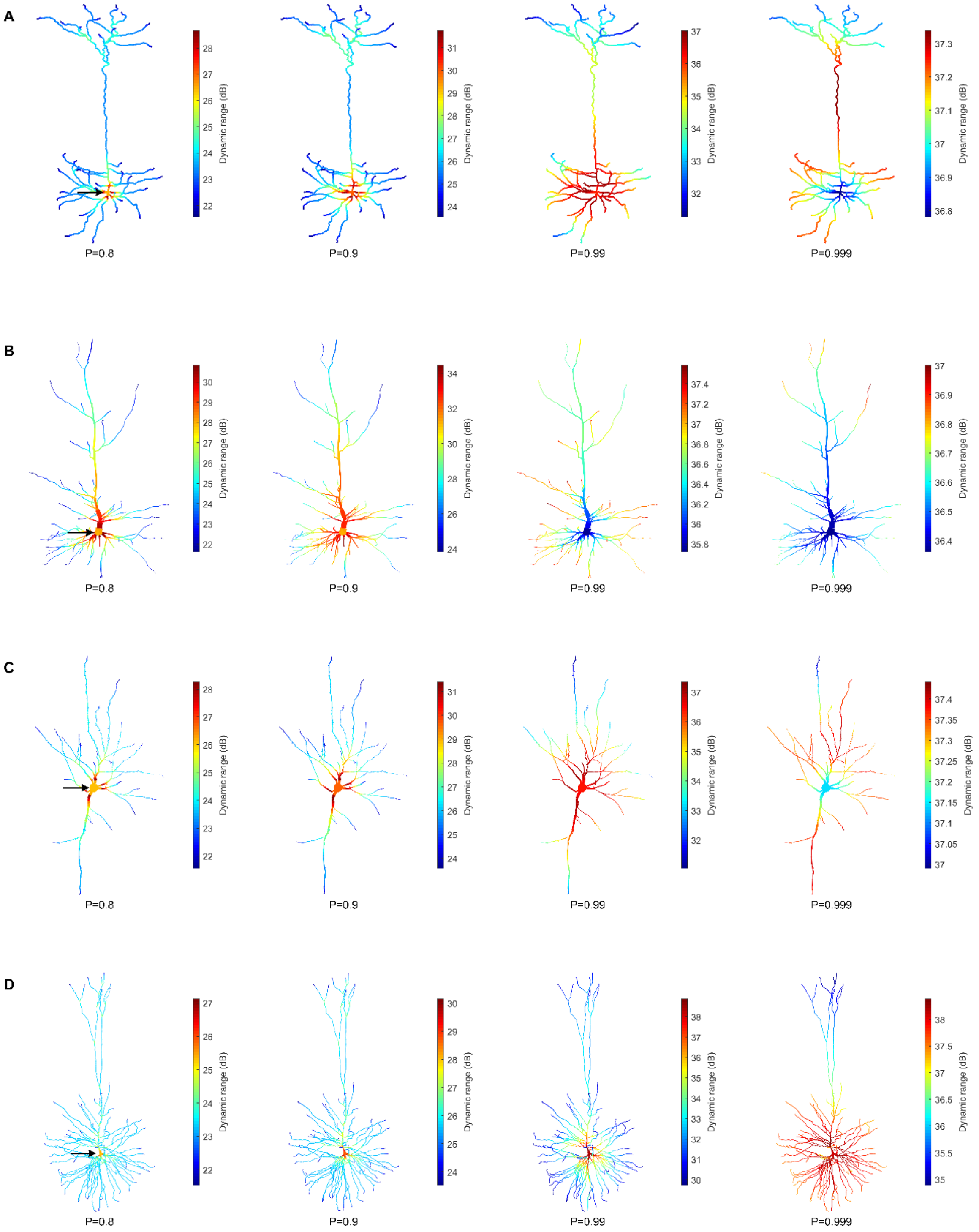

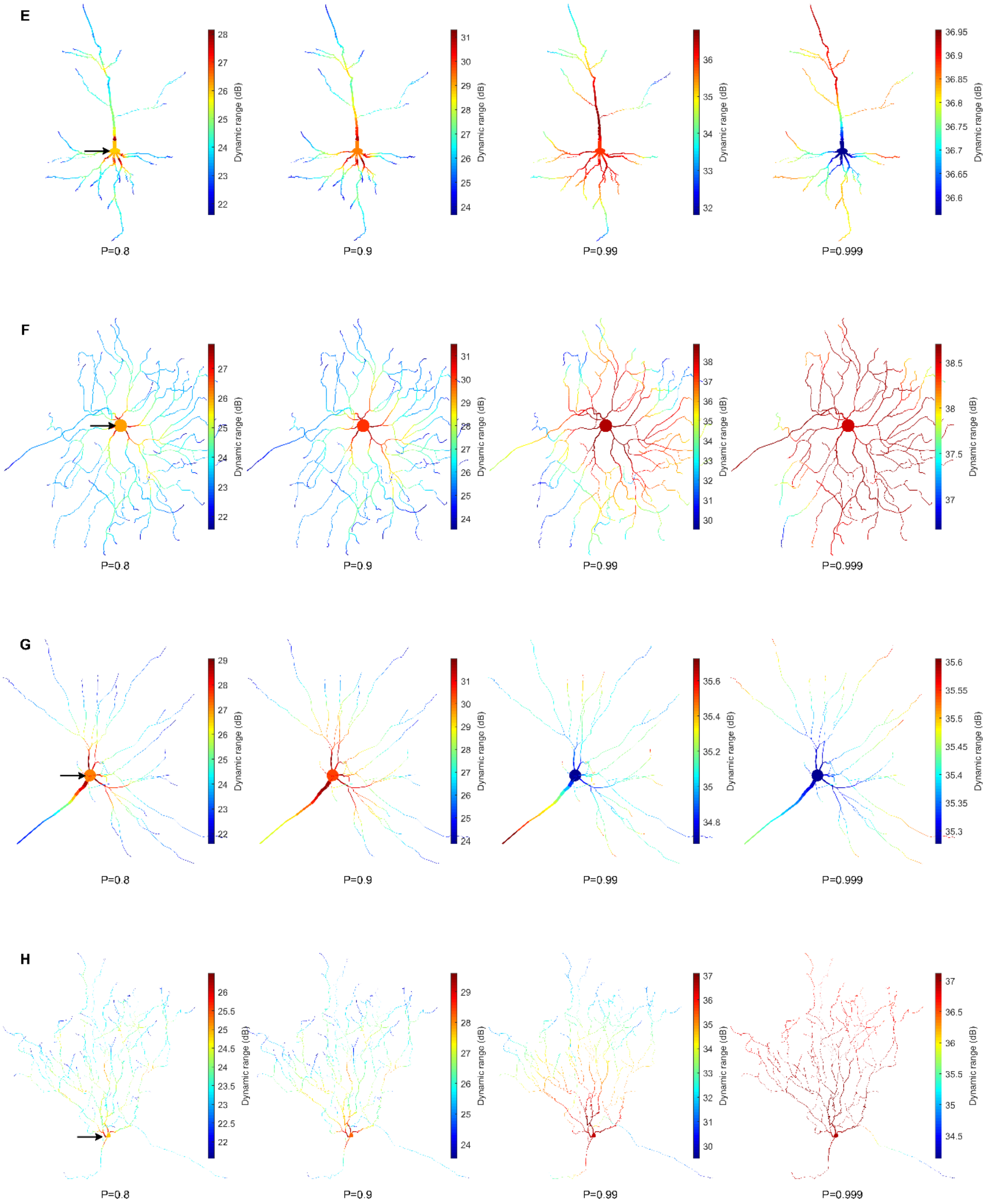

E
F
G
H
P=0.8
P=0.9
P=0.99
P=0.999
Dynamic range (dB)

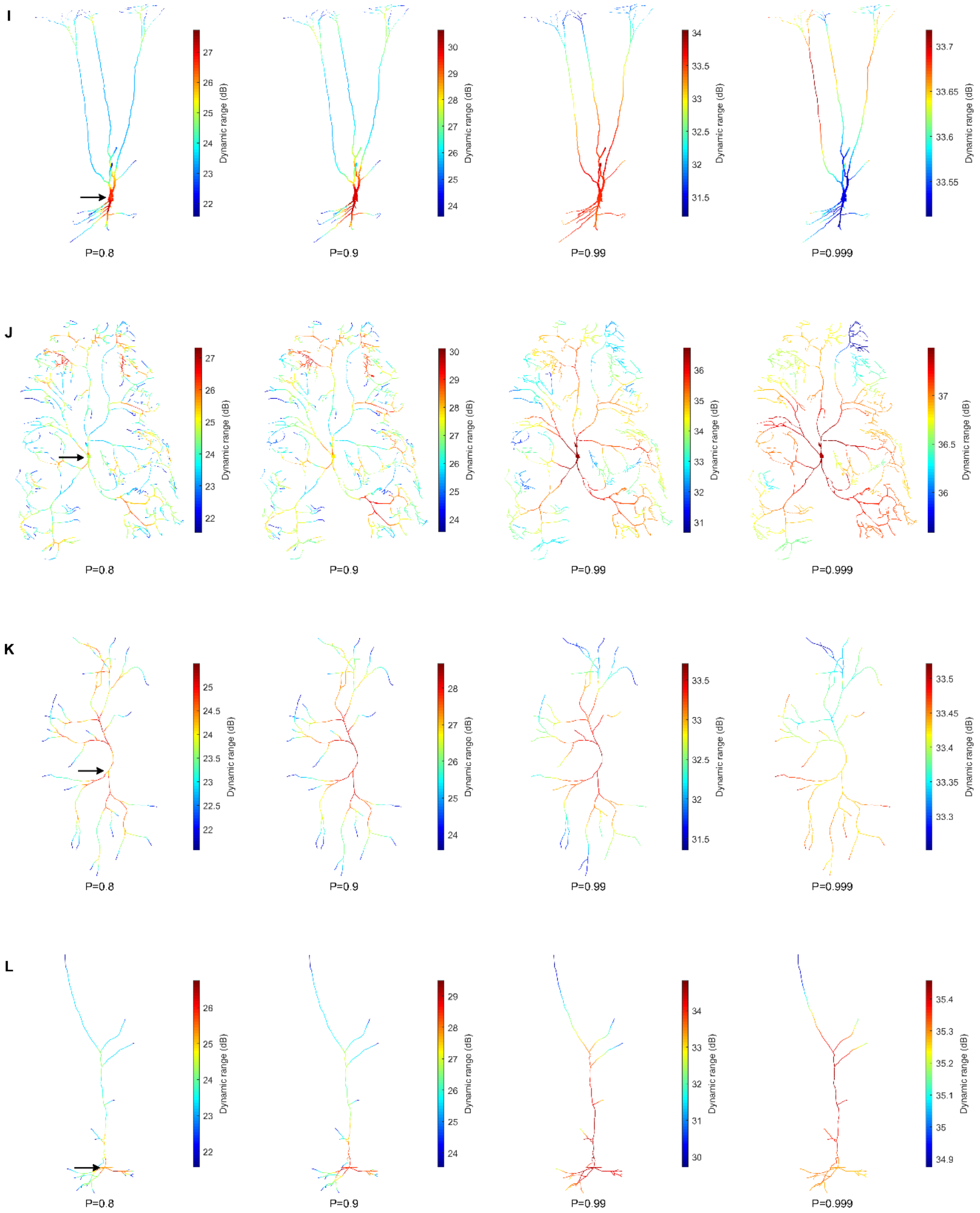
I
27 26 25 24 23 22
Dynamic range (dB)
P=0.8
30 29 28 27 26 25 24
Dynamic range (dB)
P=0.9
34 33.5 33 32.5 32 31.5
Dynamic range (dB)
P=0.99
33.7 33.65 33.6 33.55
Dynamic range (dB)
P=0.999
J
27 26 25 24 23 22
Dynamic range (dB)
P=0.8
30 29 28 27 26 25 24
Dynamic range (dB)
P=0.9
36 35 34 33 32 31
Dynamic range (dB)
P=0.99
37 36.5 36
Dynamic range (dB)
P=0.999
K
25 24.5 24 23.5 23 22.5 22
Dynamic range (dB)
P=0.8
28 27 26 25 24
Dynamic range (dB)
P=0.9
33.5 33 32.5 32 31.5
Dynamic range (dB)
P=0.99
33.5 33.45 33.4 33.35 33.3
Dynamic range (dB)
P=0.999
L
26 25 24 23 22
Dynamic range (dB)
P=0.8
29 28 27 26 25 24
Dynamic range (dB)
P=0.9
34 33 32 31 30
Dynamic range (dB)
P=0.99
35.4 35.3 35.2 35.1 35 34.9
Dynamic range (dB)
P=0.999

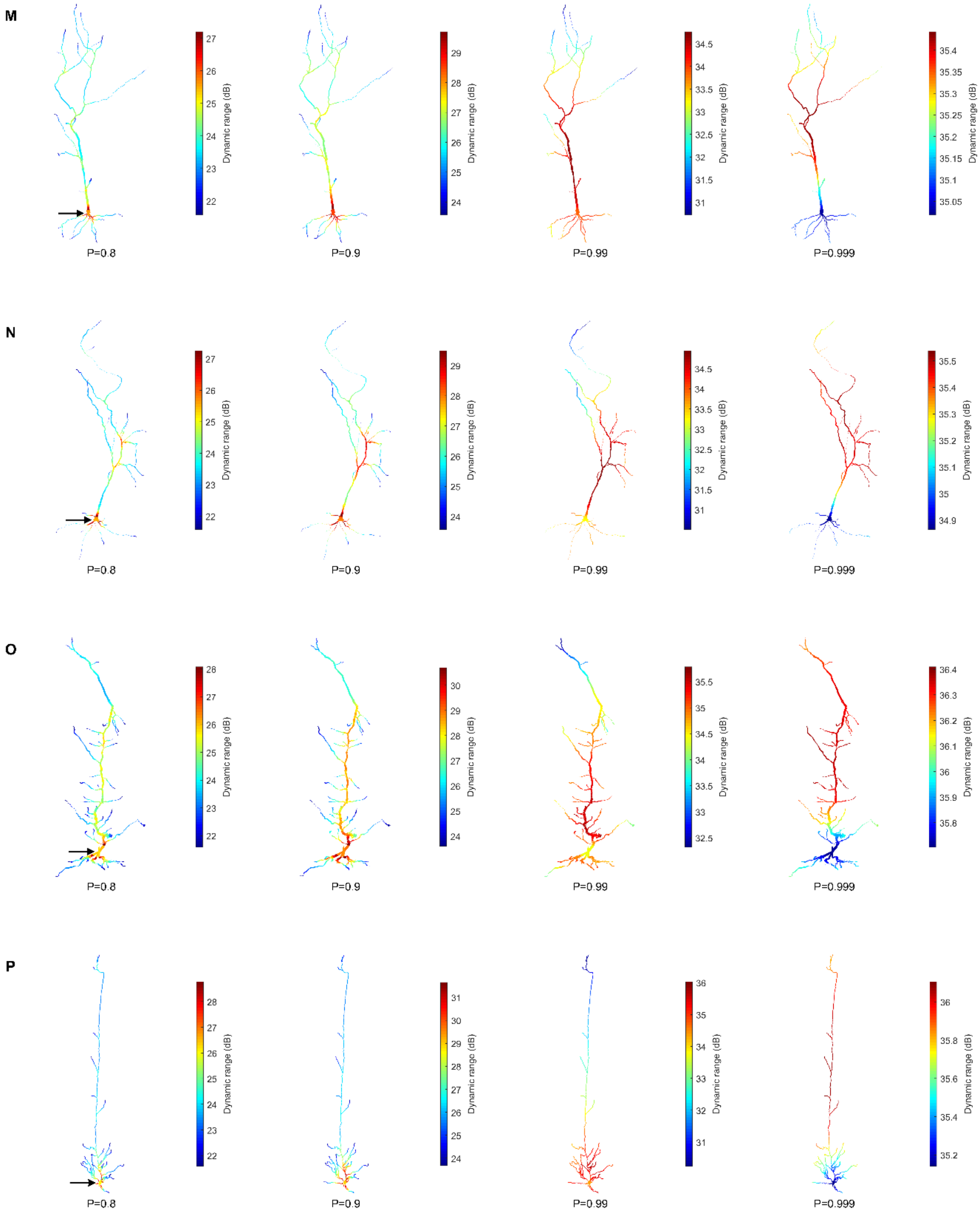
M
P=0.8
P=0.9
P=0.99
P=0.999
Dynamic range (dB)
27 26 25 24 23 22
29 28 27 26 25 24
34.5 34 33.5 33 32.5 32 31.5 31
35.4 35.35 35.3 35.25 35.2 35.15 35.1 35.05
N
P=0.8
P=0.9
P=0.99
P=0.999
Dynamic range (dB)
27 26 25 24 23 22
29 28 27 26 25 24
34.5 34 33.5 33 32.5 32 31.5 31
35.5 35.4 35.3 35.2 35.1 35 34.9
O
P=0.8
P=0.9
P=0.99
P=0.999
Dynamic range (dB)
28 27 26 25 24 23 22
30 29 28 27 26 25 24
35.5 35 34.5 34 33.5 33 32.5
36.4 36.3 36.2 36.1 36 35.9 35.8
P
P=0.8
P=0.9
P=0.99
P=0.999
Dynamic range (dB)
28 27 26 25 24 23 22
31 30 29 28 27 26 25 24
36 35 34 33 32 31
36 35.8 35.6 35.4 35.2

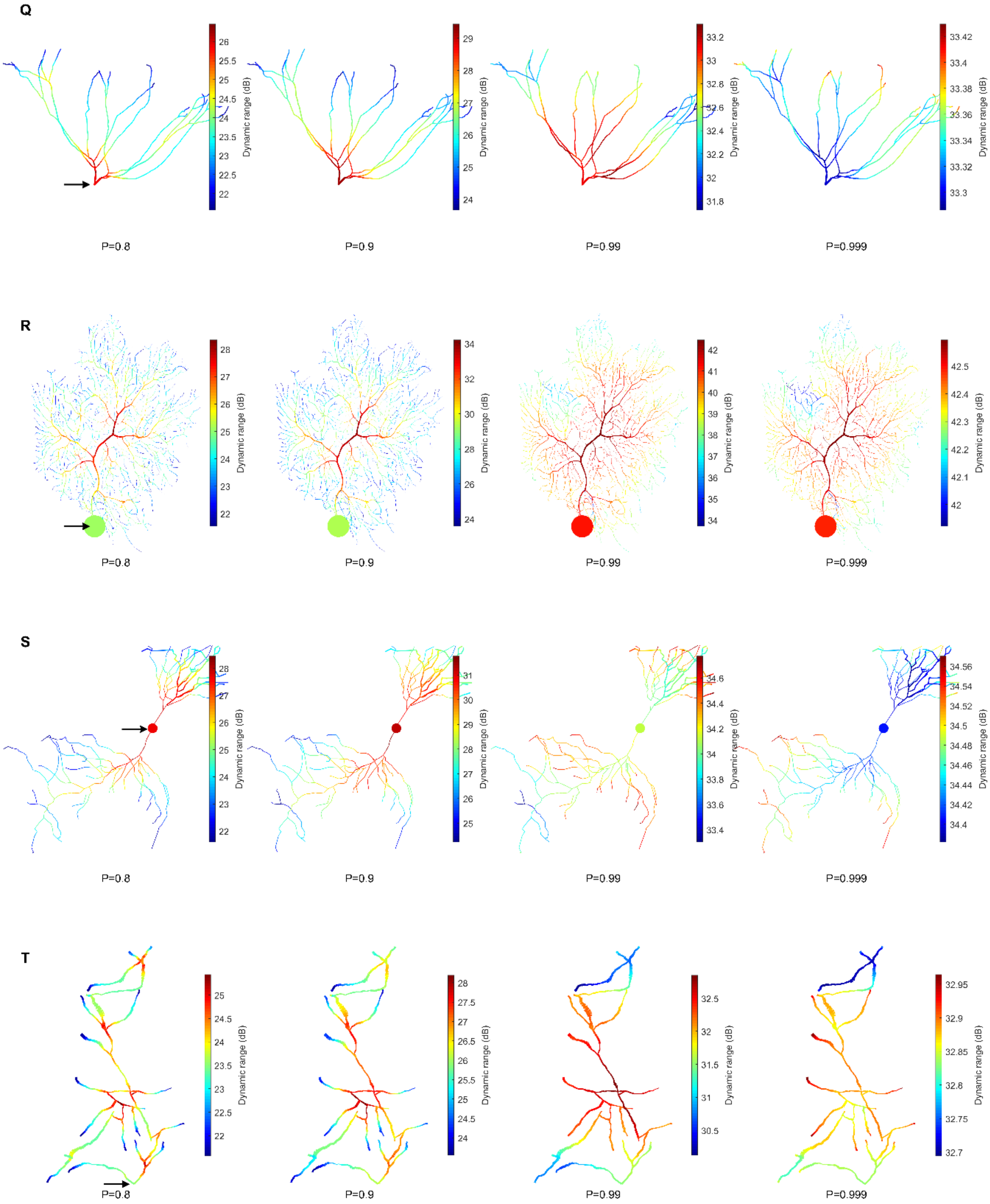

Q
Dynamic range (dB)
P=0.8
P=0.9
P=0.99
P=0.999
R
Dynamic range (dB)
P=0.8
P=0.9
P=0.99
P=0.999
S
Dynamic range (dB)
P=0.8
P=0.9
P=0.99
P=0.999
T
Dynamic range (dB)
P=0.8
P=0.9
P=0.99
P=0.999

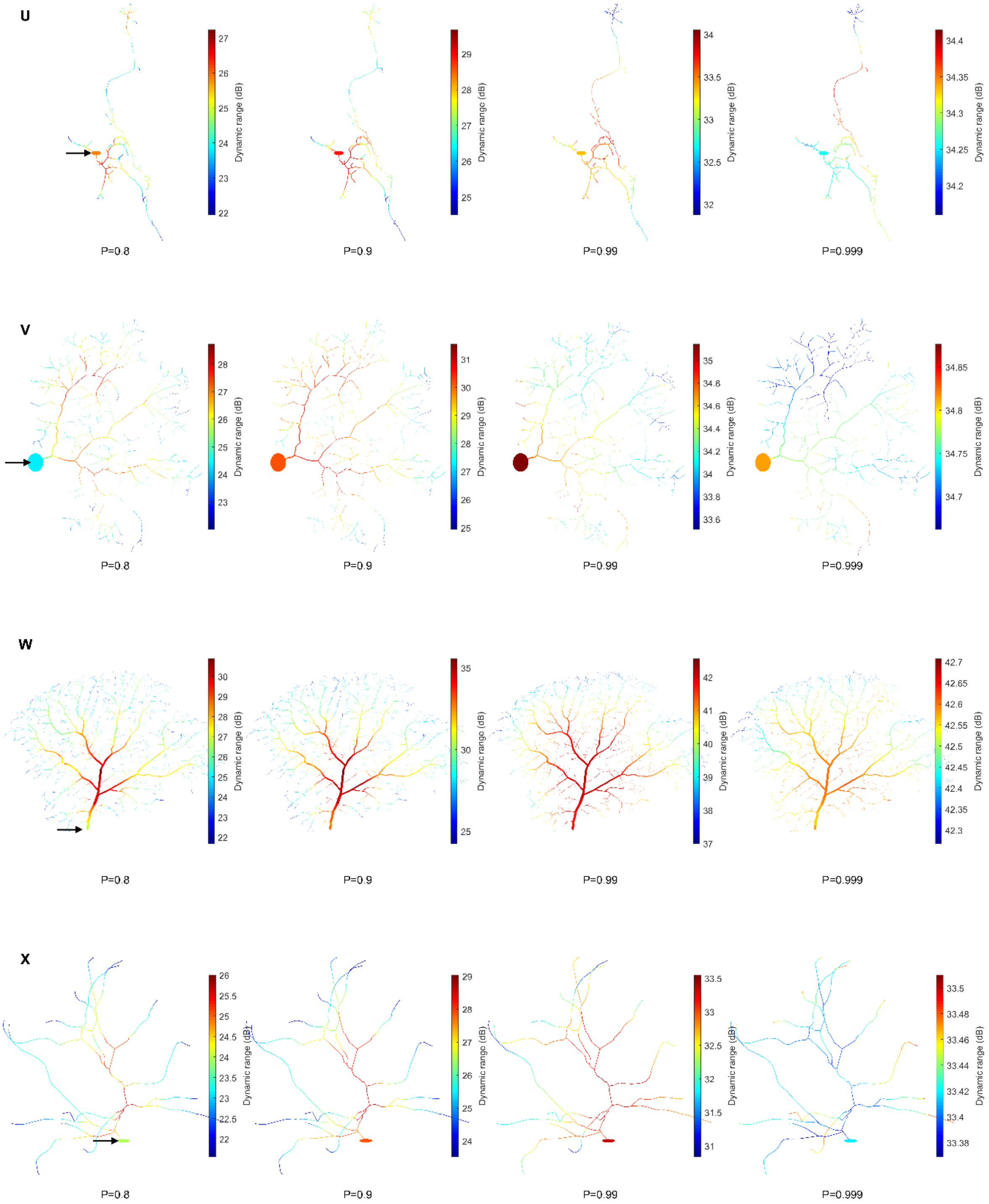

U
V
W
X
Dynamic range (dB)
P=0.8
P=0.9
P=0.99
P=0.999

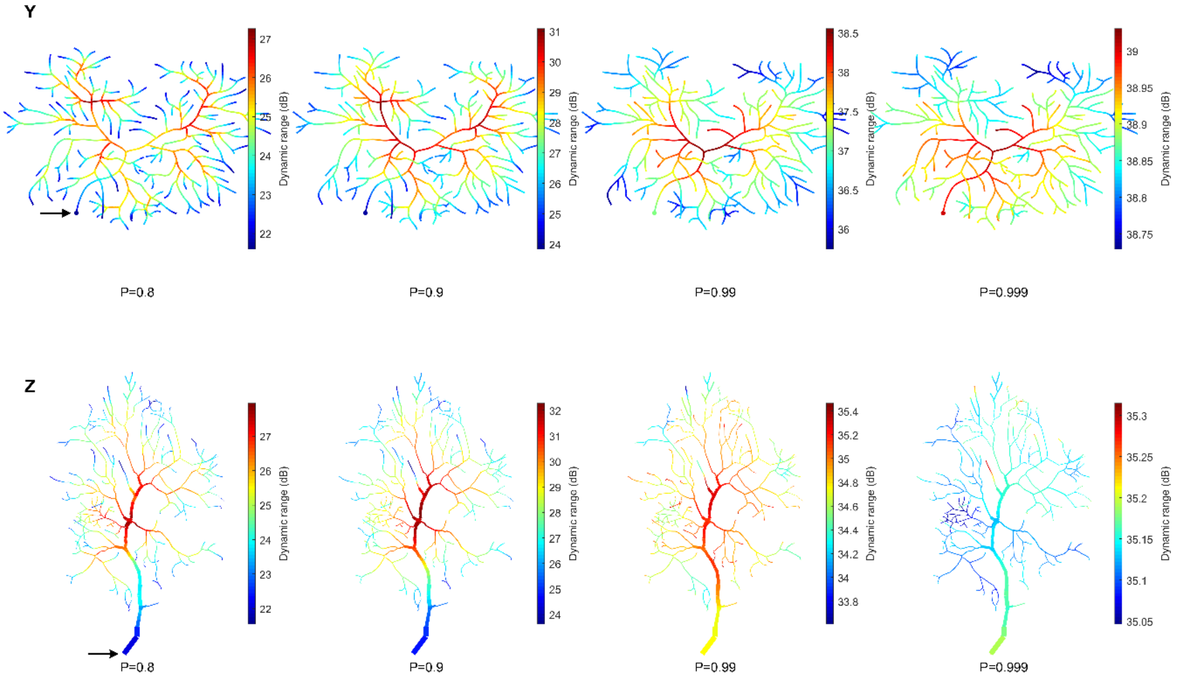

Figure S4. Compartmental dynamic range as a function of distance from soma for all neurons. See methods for description of the neurons.

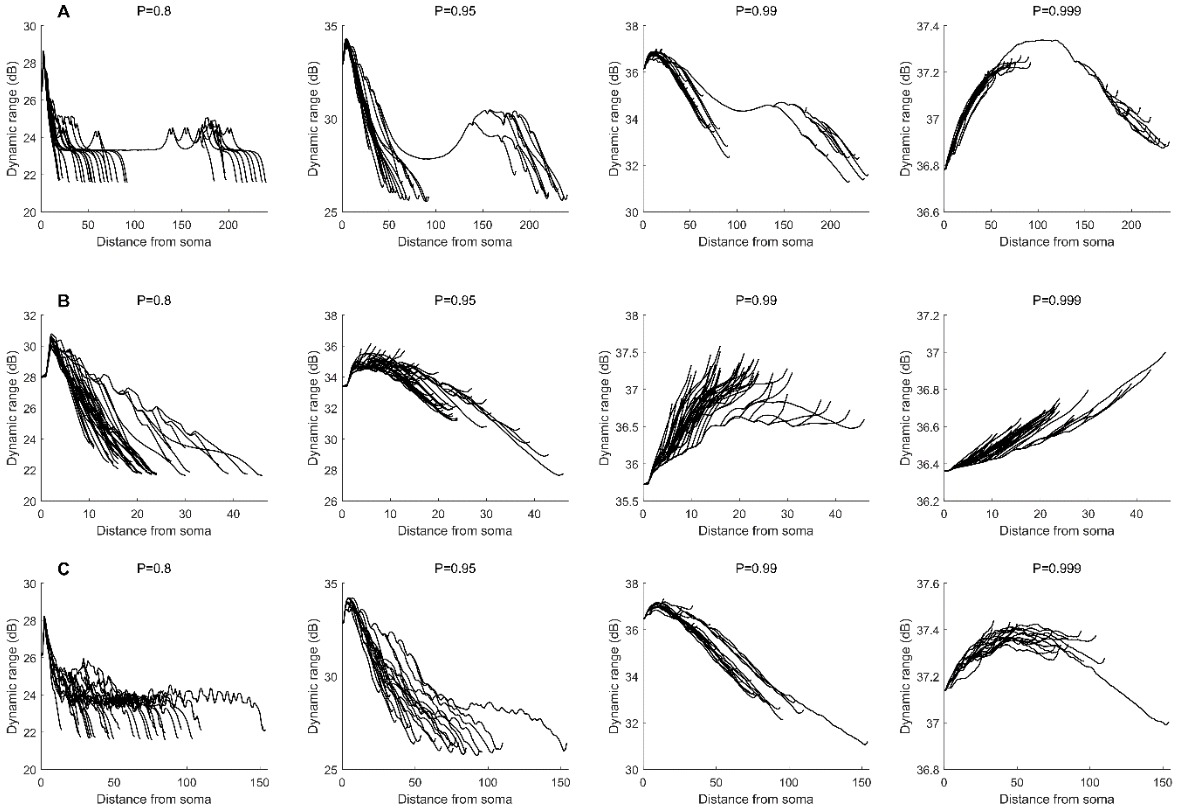

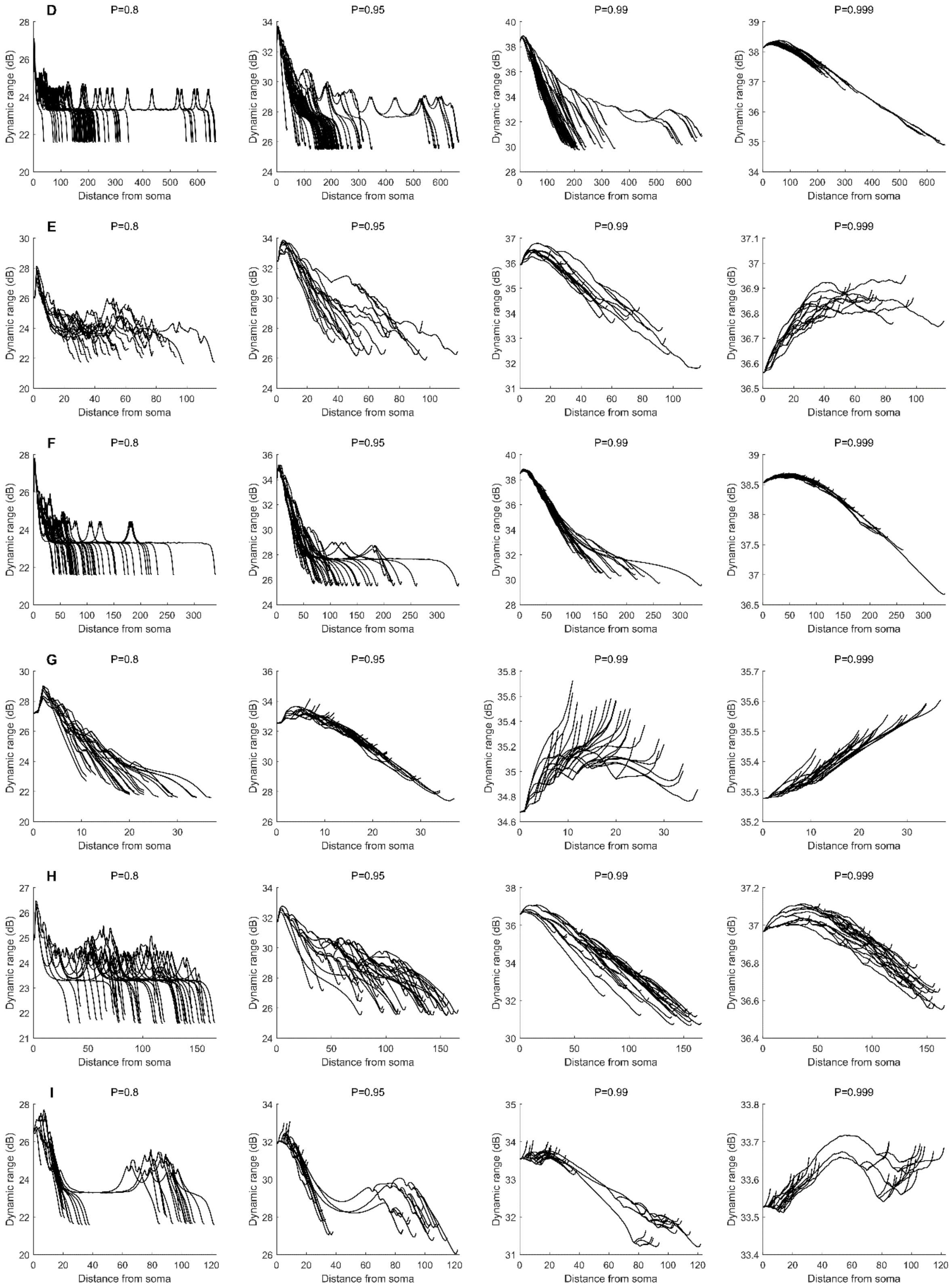
D
E
F
G
H
I
P=0.8
P=0.95
P=0.99
P=0.999
Dynamic range (dB)
Distance from soma

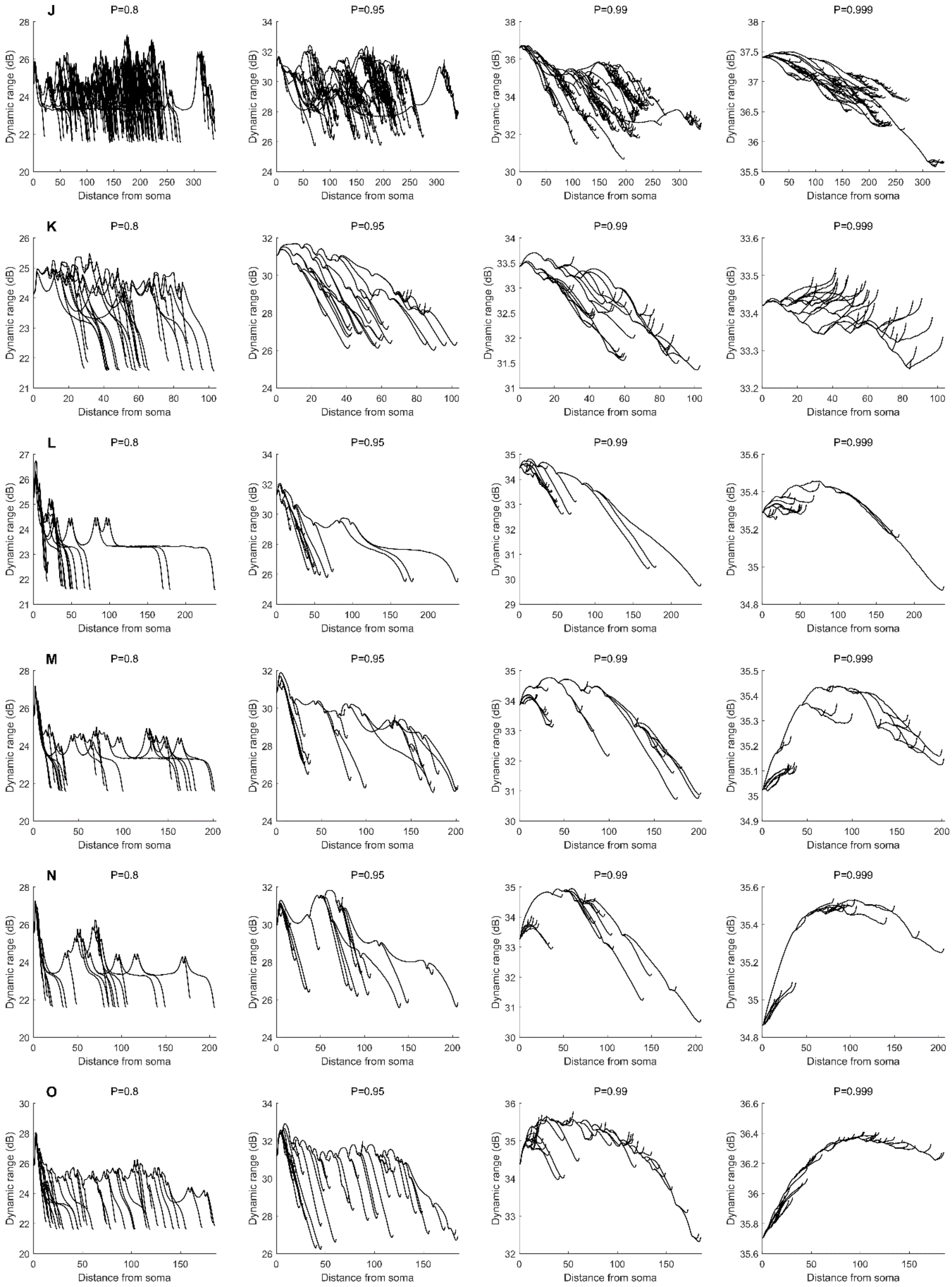
J
K
L
M
N
O
P=0.8
P=0.95
P=0.99
P=0.999
Dynamic range (dB)
Distance from soma

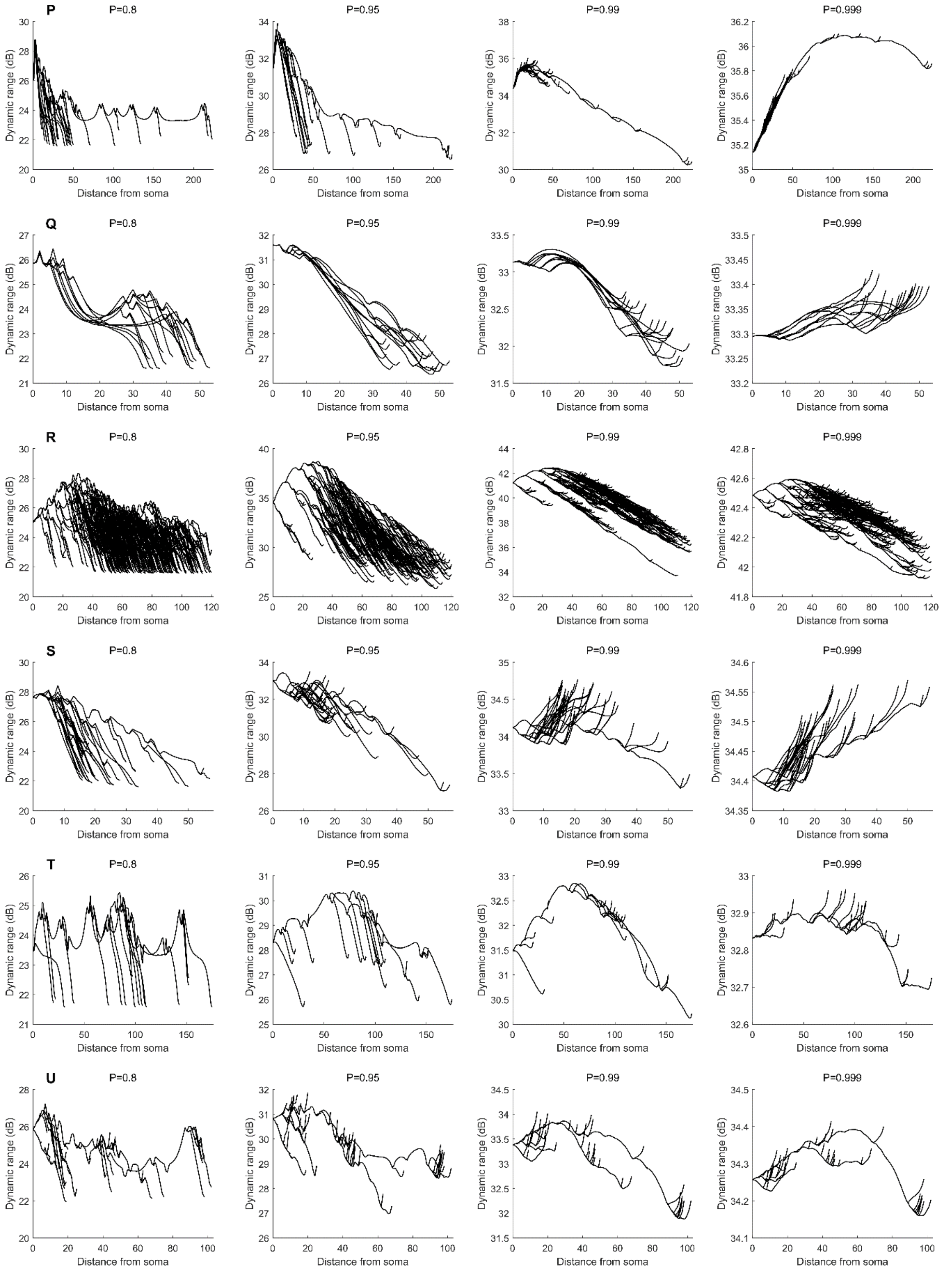

P
Q
R
S
T
U
P=0.8
P=0.95
P=0.99
P=0.999
Dynamic range (dB)
Distance from soma

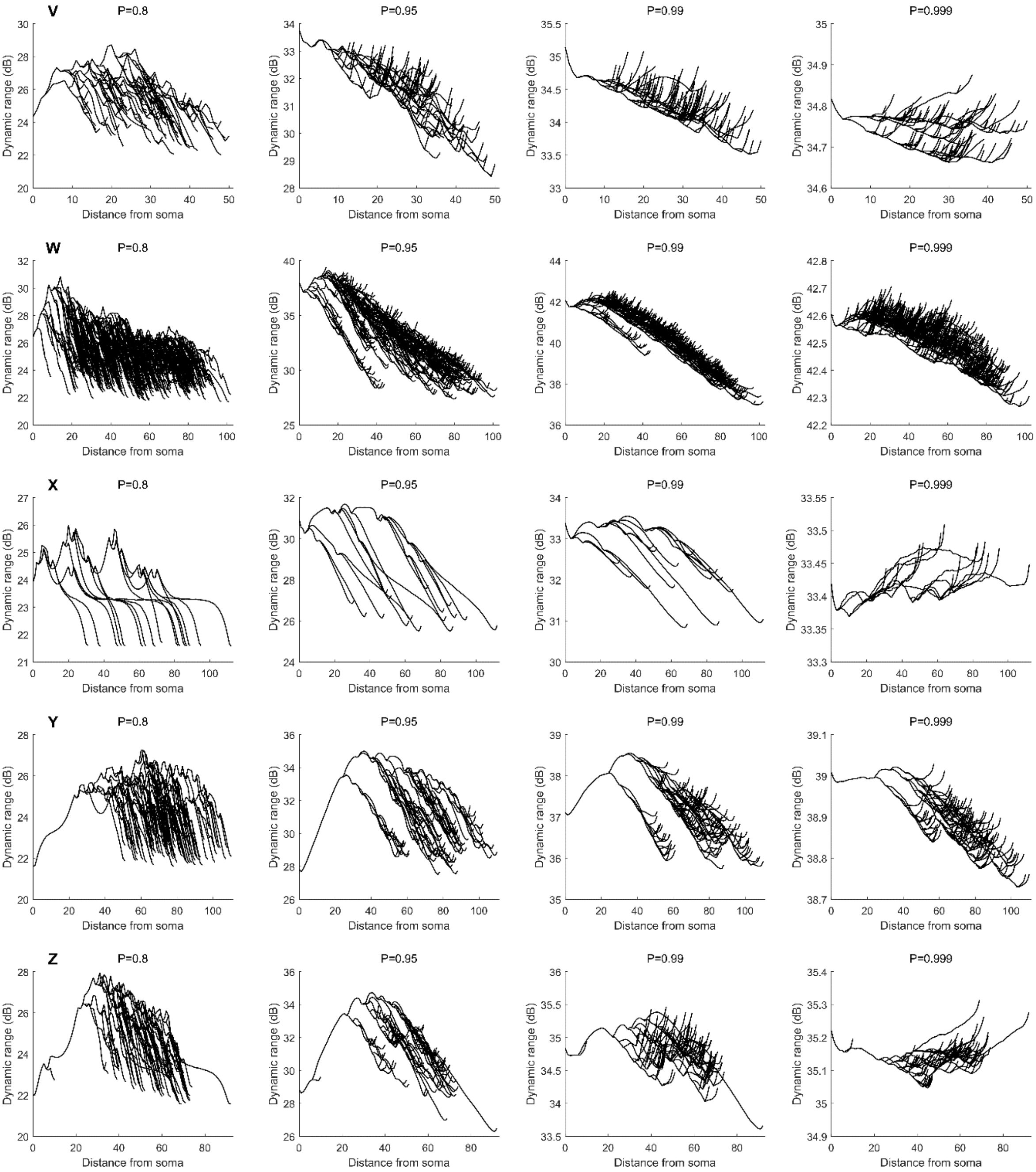
V
P=0.8
P=0.95
P=0.99
P=0.999
W
P=0.8
P=0.95
P=0.99
P=0.999
X
P=0.8
P=0.95
P=0.99
P=0.999
Y
P=0.8
P=0.95
P=0.99
P=0.999
Z
P=0.8
P=0.95
P=0.99
P=0.999
Dynamic range (dB)
Distance from soma

Figure S5. Additional dynamics of benchmark neurites. The main branch consists of N = 1000 compartments. The value of P is constant for each collection of sub-plots, and plots are arranged according to the position (Q) and length (L) of the secondary branch. In each small sub-plot, the compartmental dynamic range (y axis, in dB) is plotted against the distance from the soma (x axis), with shadings indicating ± 1 standard deviation from the mean over 10 trials. Each trial was run for 10^5 time steps (ms). As P increases, the amplitude and extent of the spike in dynamic range at the bifurcation point increases. However, for large P, short secondary branches do not have a significant effect on the dynamic range.

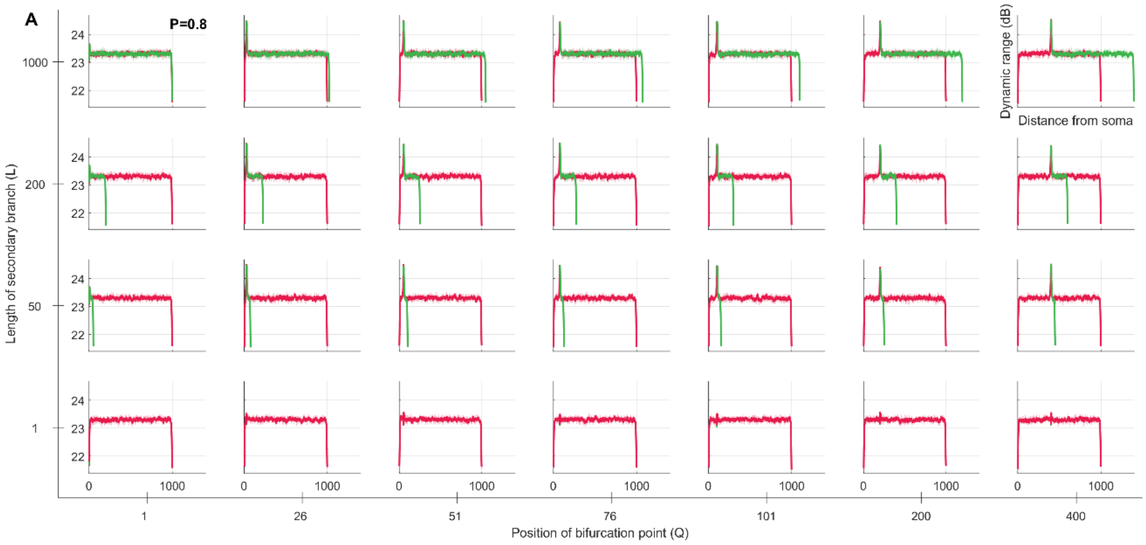

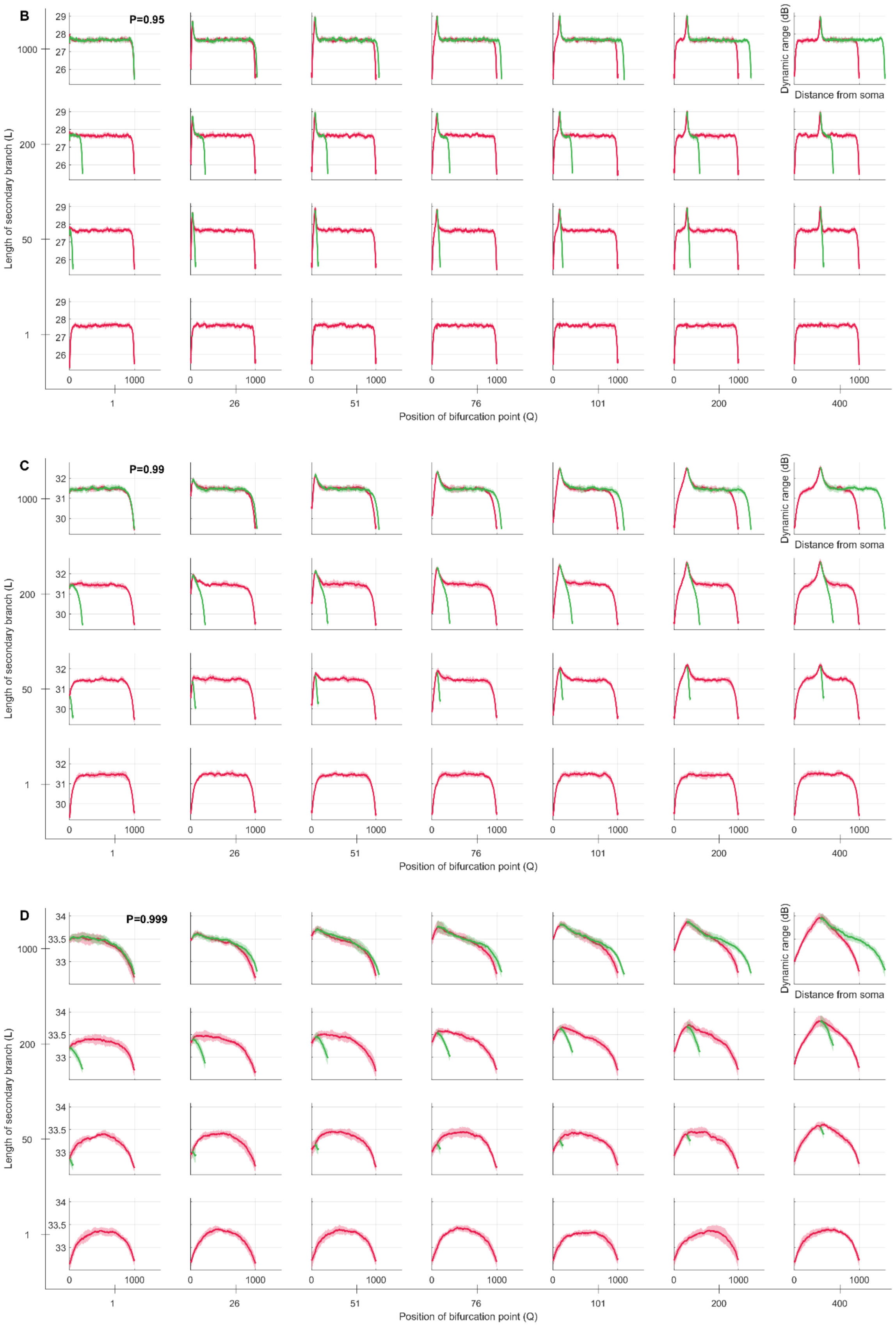
B
P=0.95
C
P=0.99
D
P=0.999
Length of secondary branch (L)
Position of bifurcation point (Q)
Dynamic range (dB)
Distance from soma
1000
200
50
1
26
51
76
101
400
29
28
27
26
32
31
30
34
33.5
33
0

Figure S6. Relative energy consumption plots of all neurons. The energy consumption is a measure of how often each dendritic spike fires per somatic spike (see Methods). There are several distinct behaviors, which we attribute to the number of branches connected to the soma, and the relative centrality of the soma. Note the different color bars.

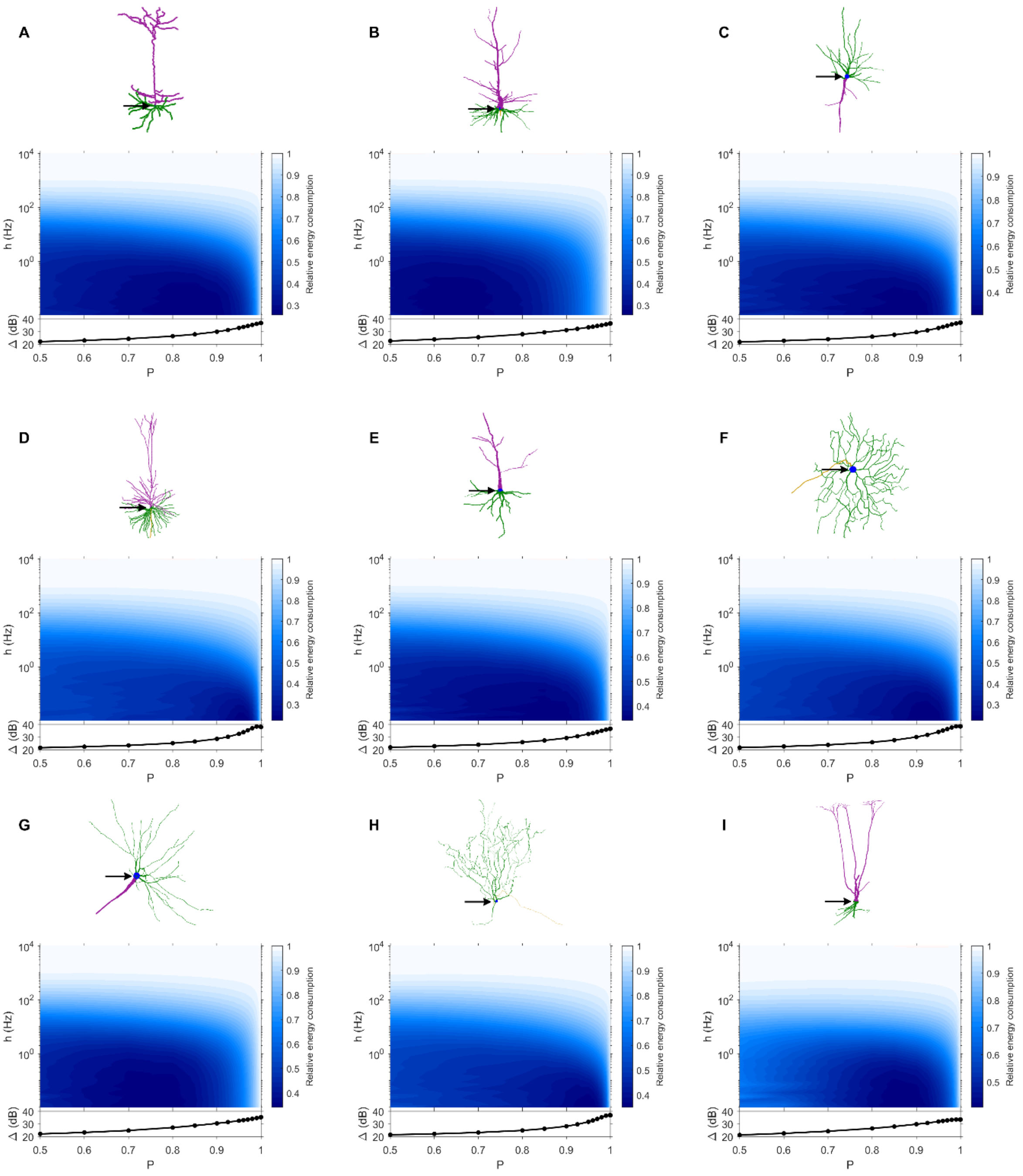

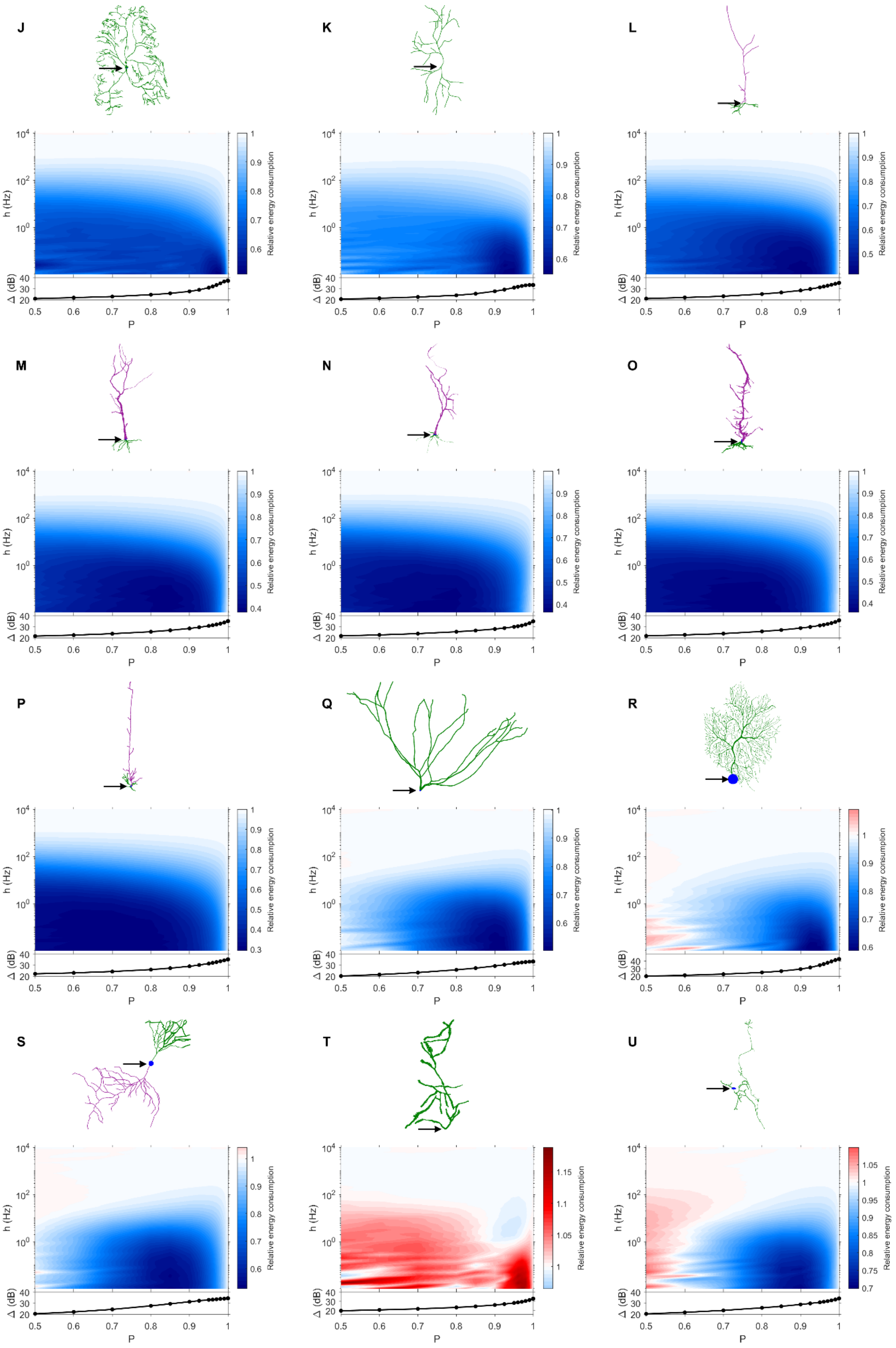
J
K
L
M
N
O
P
Q
R
S
T
U
h (Hz)
Δ (dB)
P
Relative energy consumption

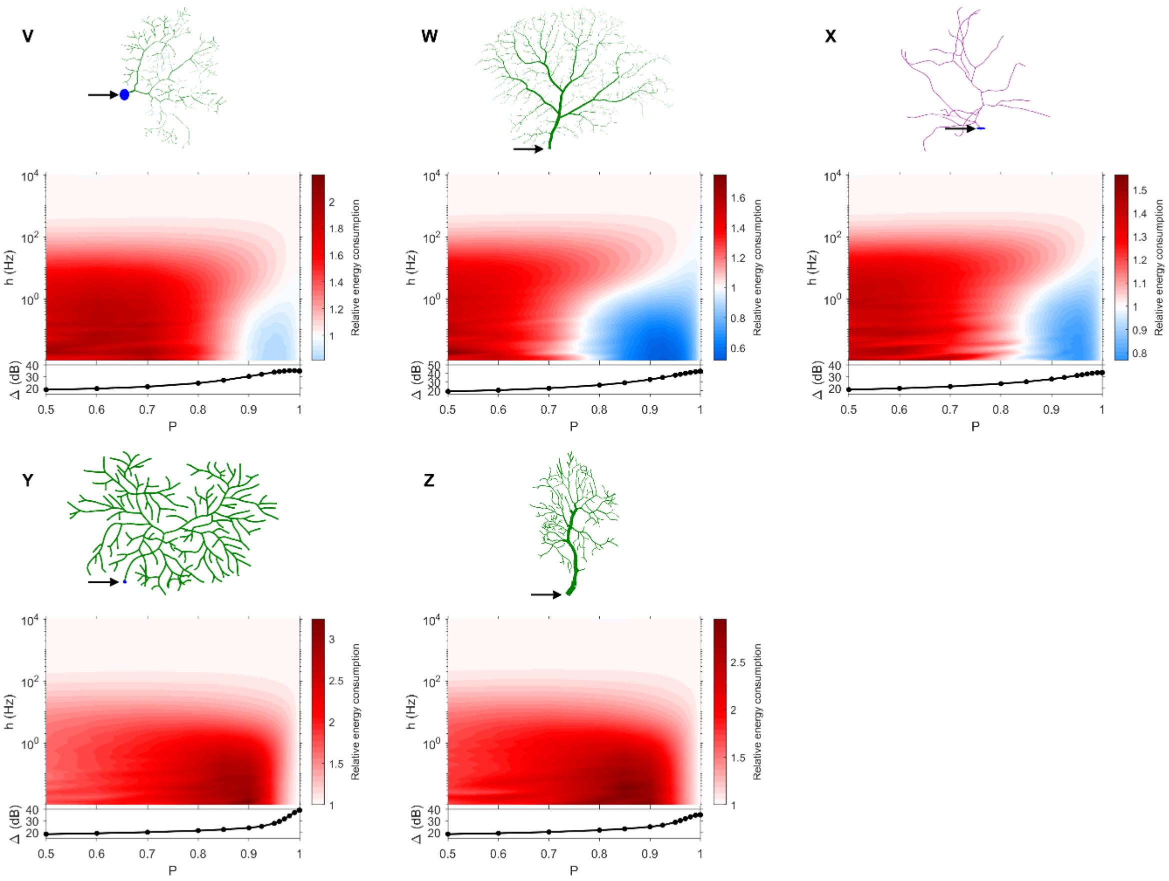

Figure S7. Relative centrality heat maps for all neurons. Red corresponds to the most central compartments ($C = 1$), while blue corresponds to the least central ones ($C = 0$). See Methods for details. The location of the soma is indicated by the arrow.

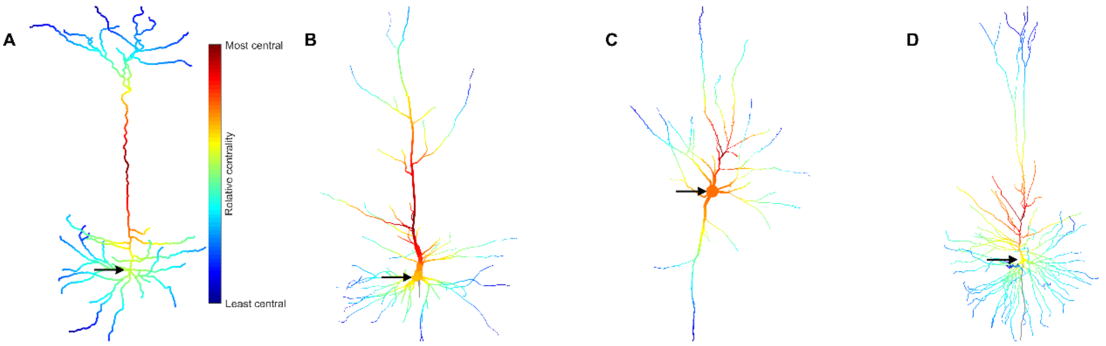

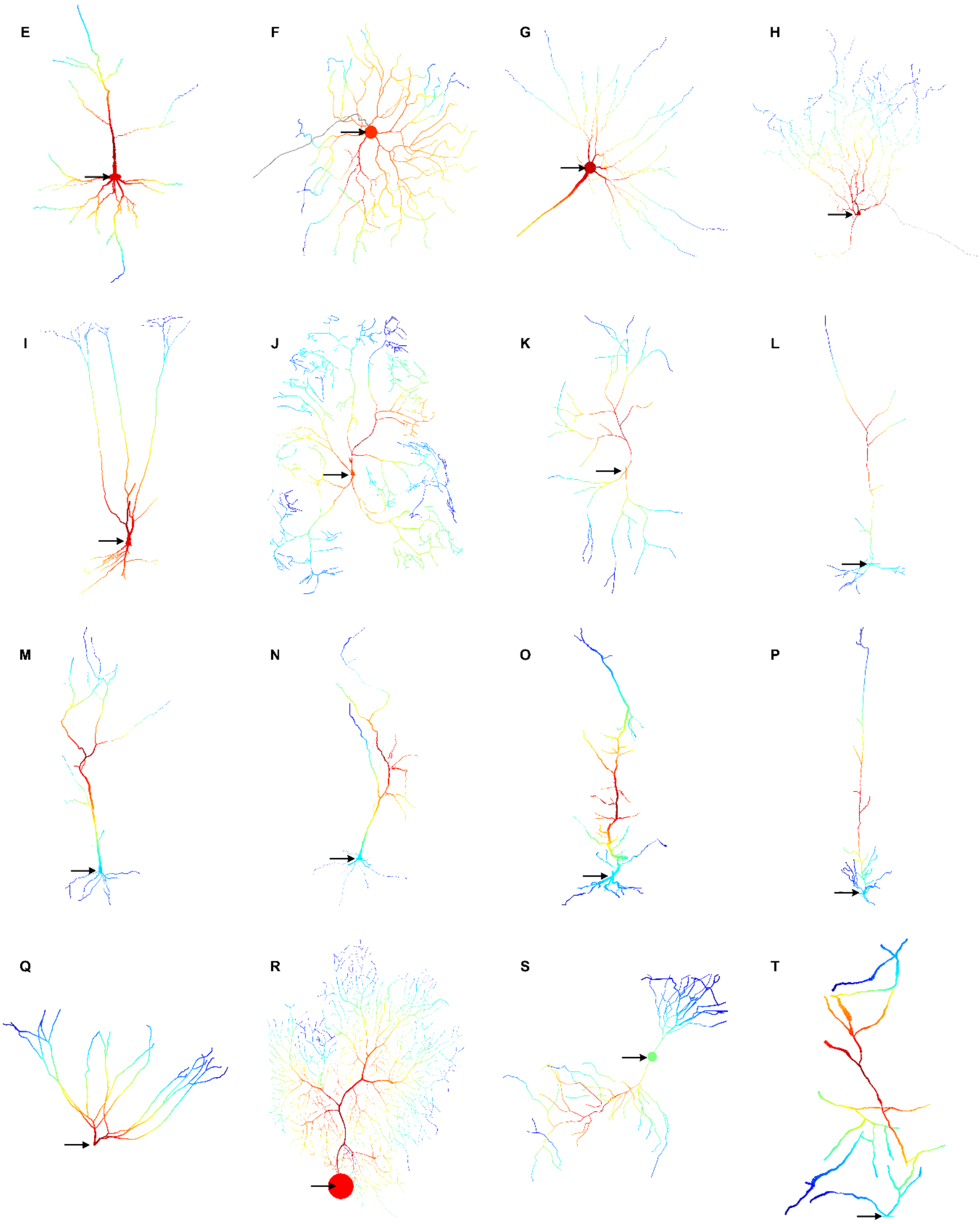
E
F
G
H
I
J
K
L
M
N
O
P
Q
R
S
T

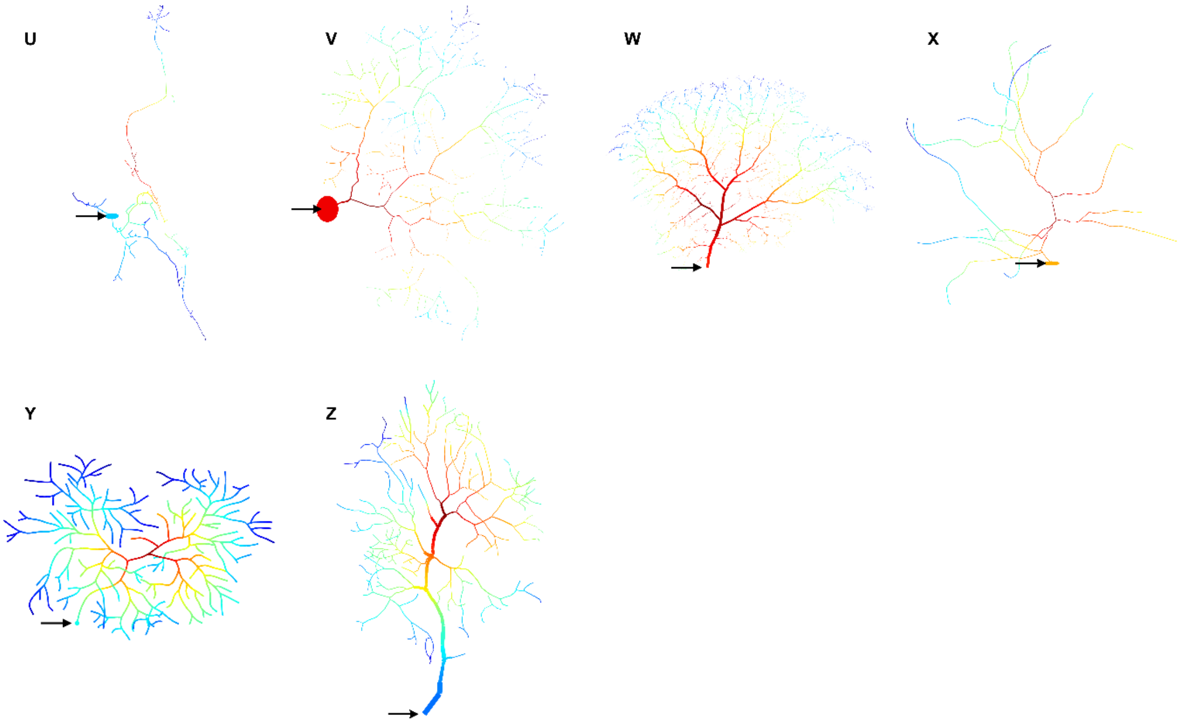
U
V
W
X
Y
Z

Figure S8. Correlation between centrality and dynamic range. Scatter plot of relative compartmental centrality (see Methods) vs relative compartmental dynamic range for all compartments in all neurons A-Z (total number of compartments is N = 47411). The relative dynamic range is Δ_{rel} = 1 if a compartment has the largest dynamic range in the neuron, and Δ_{rel} = 0 if it has the lowest. The soma of each neuron is marked in red.

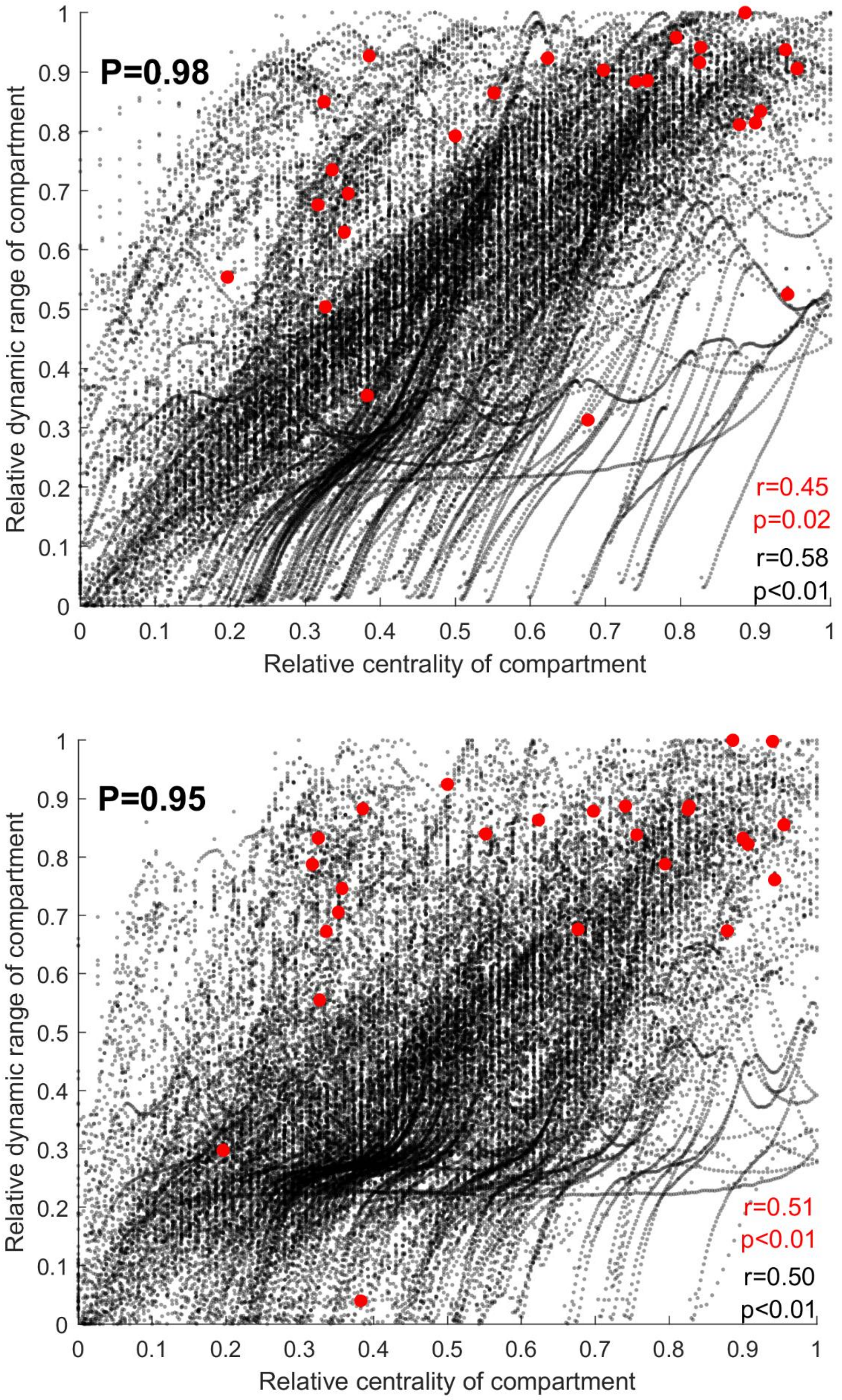

Supplement: Supplemental Information 2 [file peerj-08-10250-s002.pdf]
